# Gut-dependent microbial translocation induces inflammation and cardiovascular events after ST-elevation myocardial infarction

**DOI:** 10.1186/s40168-018-0441-4

**Published:** 2018-04-03

**Authors:** Xin Zhou, Jing Li, Junli Guo, Bin Geng, Wenjie Ji, Qian Zhao, Jinlong Li, Xinlin Liu, Junxiang Liu, Zhaozeng Guo, Wei Cai, Yongqiang Ma, Dong Ren, Jun Miao, Shaobo Chen, Zhuoli Zhang, Junru Chen, Jiuchang Zhong, Wenbin Liu, Minghui Zou, Yuming Li, Jun Cai

**Affiliations:** 1 Tianjin Key Laboratory of Cardiovascular Remodeling and Target Organ Injury, Pingjin Hospital Heart Center, 220, Cheng-Lin Street, Tianjin, 300162 China; 2 Heart Center, Beijing Chao Yang Hospital, Capital Medical University, Beijing, 100020 China; 3 Beijing Key Laboratory of Hypertension, Beijing, 100020 China; 4 Cardiovascular Institute of Affiliated Hospital, Hainan Medical College, Haikou, 571199 China; 5 Hypertension Center, Fuwai Hospital, State Key Laboratory of Cardiovascular Disease of China, National Center for Cardiovascular Diseases of China, Chinese Academy of Medical Sciences and Peking Union Medical College, Xicheng District, North Lishi Road No. 167, Beijing, 100037 China; 6 Department of Radiology, Northwestern University, Chicago, IL 60611 USA; 7 Novogene Bioinformatics Institute, Beijing, 100000 China; 8 Eminent Scholar in Molecular Medicine, Georgia Research Alliance, Georgia State University, Atlanta, USA

**Keywords:** Myocardial infarction, Gut permeability, Microbial translocation, Cardiovascular outcome

## Abstract

**Background:**

Post-infarction cardiovascular remodeling and heart failure are the leading cause of myocardial infarction (MI)-driven death during the past decades. Experimental observations have involved intestinal microbiota in the susceptibility to MI in mice; however, in humans, identifying whether translocation of gut bacteria to systemic circulation contributes to cardiovascular events post-MI remains a major challenge.

**Results:**

Here, we carried out a metagenomic analysis to characterize the systemic bacteria in a cohort of 49 healthy control individuals, 50 stable coronary heart disease (CHD) subjects, and 100 ST-segment elevation myocardial infarction (STEMI) patients. We report for the first time higher microbial richness and diversity in the systemic microbiome of STEMI patients. More than 12% of post-STEMI blood bacteria were dominated by intestinal microbiota (*Lactobacillus*, *Bacteroides*, and *Streptococcus*). The significantly increased product of gut bacterial translocation (LPS and d-lactate) was correlated with systemic inflammation and predicted adverse cardiovascular events. Following experimental MI, compromised left ventricle (LV) function and intestinal hypoperfusion drove gut permeability elevation through tight junction protein suppression and intestinal mucosal injury. Upon abrogation of gut bacterial translocation by antibiotic treatment, both systemic inflammation and cardiomyocyte injury in MI mice were alleviated.

**Conclusions:**

Our results provide the first evidence that cardiovascular outcomes post-MI are driven by intestinal microbiota translocation into systemic circulation. New therapeutic strategies targeting to protect the gut barrier and eliminate gut bacteria translocation may reduce or even prevent cardiovascular events post-MI.

**Electronic supplementary material:**

The online version of this article (10.1186/s40168-018-0441-4) contains supplementary material, which is available to authorized users.

## Background

Myocardial infarction (MI) is one of the most important causes of heart failure and cardiac death worldwide [1]. Despite therapeutic approaches of mechanical reperfusion by percutaneous coronary intervention (PCI) have reduced the acute mortality rates of MI, a growing body of evidence suggests the incidence of cardiovascular events post-MI still predicts an increased mortality [2]. Many experimental and clinical observations have involved inflammation in post-infarction cardiovascular remodeling and heart failure [2, 3]. The inflammatory reaction triggered by MI not only initiates cardiac repair but leads to remodeling and cardiac dysfunction [2, 3]. Although little attention has been given up to identify the pathological mechanisms underlying inflammation and cardiovascular events post-MI, ideal therapeutic strategies remain sparse.

Recent studies have suggested the involvement of gut microbiota in the development of chronic diseases, such as inflammatory bowel disease, liver cirrhosis, arthritis, and type 2 diabetes [4–7]. Atherosclerosis is also demonstrated to be promoted by intestinal metagenome and microbial metabolism [8–10]. Most recently, investigators have obtained evidence that disordered gut microbial communities are linked to the susceptibility to MI in rats [11]. Besides, following experimental MI, the intestinal permeability is suggested to be elevated [12]. The gut barrier exerts to limit intestinal bacteria and toxic mediators escaping from the gut, thus avoiding a systemic inflammatory response [13]. Gut barrier breakdown leads to intestinal permeability increase, bacteria and endotoxin (LPS) translocation into the systemic circulation, and immune-inflammatory system activation [14]. A previous report noted the circulating LPS was slightly elevated in human patients with MI compared to healthy subjects, although it did not reach statistical significance, and the sample size was quite limited [15]. Some clinical studies also reported that bacteria and LPS escaping from the gut lumen enter the systemic circulation and activate the immune-inflammatory system through triggering monocyte recruitment [14, 16–18]. d-lactate is the fermentation product of gastrointestinal bacteria [19]. High blood d-lactate concentrations are result from increased production of the metabolite in the gastrointestinal tract by bacterial flora and increased intestinal permeability with d-lactate absorption into the blood [20]. And plasma d-lactate levels have been used to assess the intestinal injury and to indicate an increase in intestinal permeability in patients and rats [21–23]. Therefore, in the current study, plasmatic d-lactate was proposed as a sensitive marker in detecting gut failure due to the damaged intestinal barrier.

Based on the information, a hypothesis was established that MI drives intestinal barrier failure and gut mucosa permeability elevation causes gut bacteria and microbial products translocation into systemic circulation, which activates excessive immune inflammation and contributes to increased risk of cardiovascular events after MI. We recruited 199 human individuals of healthy control, coronary heart disease (CHD), or ST-segment elevation myocardial infarction (STEMI), characterized their systemic microbiome by metagenomic analysis, and found significantly increased gut microbiota translocation into the systemic circulation post-STEMI. For STEMI patients, the highest increase concentration of gut bacterial products (LPS and d-lactate) in day 2 compared to day 1 of symptom onset have great prognostic significance for cardiovascular outcomes during a 3-year follow-up. Upon ischemia stress post-MI, such as depressed left ventricle (LV) function and intestinal hypoperfusion, gut permeability was increased because of the loss of tight junction protein occludin and intestinal mucosal injury. Systemic inflammation and cardiomyocyte injury were reduced through inhibition of gut bacterial translocation by antibiotic treatment. This study is the first effort to identify the contribution of gut bacteria translocation to cardiovascular outcomes pathogenesis after MI and provide novel therapeutic insight in gut-targeted strategies to improve cardiovascular events in the future.

## Results

### General characteristics of study participants during a follow-up of 3 years

The study cohort in our previous work [24] consisting of 49 healthy controls, 50 stable CHDs and 100 STEMI patients were recruited for the present study. Stable CHD was defined as previous myocardial infarction (12 weeks earlier), unstable angina (6 weeks earlier), evidence of coronaryartery disease by arteriography, or a revascularization procedure [25].

As shown in Table 1, compared to healthy controls and stable CHD, STEMI patients presented with higher admission glucose levels, increase of LPS, d-lactate and high-sensitivity C-reactive protein (hs-CRP), lower high-density lipoprotein levels, and compromised left ventricular ejection fraction (LVEF). We tested hs-CRP level on day 2 because a previous report on hs-CRP dynamic changes after STEMI showed that the peak level was observed 48 to 72 h after STEMI [26]. There was a significant difference in lymphocyte, monocyte, and neutrophil counts between healthy controls and STEMI (Table 1).

**Table 1:** Characteristics of controls, stable CHD, and STEMI patients during a median follow-up of 3 years

	Healthy controls	Stable CHD	STEMI	STEMI (*n* = 100)		
	(*n* = 49)	(*n* = 50)	(*n* = 100)	MACE (+) (*n* = 33)	MACE (−) (*n* = 67)	*P* value
Gender (female)	15 (30.6%)	13 (26.0%)	22 (22.0%)	6 (18.2%)	16 (23.9%)	0.613
Age (year)	59.4 ± 8.96	61.7 ± 8.82	59.5 ± 11.4	61.4 ± 10.9	58.6 ± 11.6	0.264
Body mass index (kg/m^2^)	25.0 ± 3.1	24.7 ± 3.1	24.9 ± 3.6	24.5 ± 3.1	25.0 ± 4.0	0.561
Current smoking	25 (51.0%)	27 (54.0%)	68 (68.0%)	22 (6.1%)	46 (68.7%)	1.000
Hypertension	0	29 (58%.0)*	54 (54.0%)*	19 (57.6%)	35 (52.2%)	0.673
Diabetes	0	14 (28.0%)*	22 (22.0%)*	9 (27.3%)	13 (19.4%)	0.443
Door-to-balloon time (min)	N/A	N/A	67.1 ± 9.0	67.0 ± 7.8	67.2 ± 9.6	0.932
Symptom-to-admission time (h)	N/A	N/A	4.0 (2.1, 6.0)	4.0 (2.5, 6.5)	4.0 (2.0, 6.0)	0.691
Infarct region (anterior MI)	N/A	N/A	48 (48.0%)	21 (63.6%)	27 (40.3%)	*0.034* ^#^
Blood biochemical tests						
Troponin T_admission (ng/ml)	N/A	N/A	1.03 (0.71, 1.49)	0.99 (0.68, 1.58)	1.03 (0.78, 1.43)	0.570
Troponin T_peak (ng/ml)	N/A	N/A	1.22 (0.89, 1.63)	1.22 (0.81, 1.76)	1.22 (0.92, 1.61)	0.782
eGFR (ml/min/1.73 m^2^)	105 ± 18	103 ± 24	107 ± 32	106 ± 36	107 ± 30	0.912
Glucose (mmol/l)	5.0 (4.6, 5.4)	5.6 (4.9, 6.2)*	6.2 (5.5, 8.1)*†	6.8 (5.4, 10.0)	6.1 (5.5, 7.2)	0.136
Total cholesterol (mmol/l)	4.1 (3.7, 5.0)	4.6 (3.7, 5.5) *	4.5 (4.0, 5.0) *	4.5 (4.0, 4.8)	4.6 (4.0, 5.1)	0.363
Triglyceride (mmol/l)	1.4 (1.0, 1.8)	1.3 (0.9, 2.0)	1.5 (1.0, 2.0)	1.4 (0.9, 1.9)	1.5 (1.0, 2.0)	0.294
High-density lipoprotein (mmol/l)	1.4 (1.1, 1.5)	1.4 (1.2, 1.7)	1.2 (1.0, 1.3) *†	1.2 (1.0, 1.4)	1.2 (1.1, 1.3)	0.907
Low-density lipoprotein (mmol/l)	2.2 (2.0, 2.7)	2.5 (2.0, 2.8)	2.3 (2.0, 2.6)	2.2 (1.8, 2.5)	2.3 (2.0, 2.7)	0.109
LVEF (%, Echocardiography)	60 (57, 63)	60 (56, 62)	48 (43, 55) *†	46 (36, 51)	53 (45, 55)	*0.001* ^#^
SYNTAX score	N/A	N/A	19 (11, 22)	20 (15, 26)	18 (9, 22)	*0.028* ^#^
Blood routine tests						
White blood cell (10^9^/l)	5.95 (5.33, 6.73)	6.14 (5.10, 7.00)	10.4 (8.27, 12.7) *†	10.0 (8.0, 13.6)	10.4 (8.3, 12.7)	0.889
Lymphocyte (10^9^/l)	2.04 (1.60, 2.70)	1.68 (1.37, 2.15) ^*^	1.60 (1.20, 2.47) *	1.80 (1.17, 2.49)	1.60 (1.20, 2.46)	0.860
Monocyte (10^9^/l)	0.39 (0.27, 0.47)	0.37 (0.30, 0.42)	0.50 (0.40, 0.65) *†	0.50 (0.40, 0.70)	0.50 (0.39, 0.60)	0.491
Neutrophil (10^9^/l)	3.36 (2.95, 3.95)	3.90 (2.97, 4.61)	7.75 (5.81, 10.31) *†	8.1 (5.3, 10.5)	7.4 (5.8, 10.2)	0.924
Plasma LPS (endotoxin unit/ml)	0.44 (0.30, 0.56)	0.48 (0.33, 0.60)	1.03 (0.83, 1.39) *†	1.05 (0.85, 1.40)	1.02 (0.81, 1.37)	0.664
Plasma d-lactate (mg/l)	13.1 (10.5, 15.8)	14.5 (12.2, 17.2)	29.3 (21.4, 39.5) *^†^	33.5 (27.2, 45.8)	28.1 (19.3, 33.1)	0.017
hs-CRP (mg/l)	0.59 (0.44, 0.80)	0.66 (0.45, 1.25)*	2.72 (1.79, 4.57) *^†^	4.52 (2.70, 4.95)	2.42 (1.37, 3.76)	< 0.001^#^

The blood tests and measurements were performed on day 1 of STEMI onset. LPS, d-lactate and hs-CRP measurements were carried out on day 2 of symptom onset

*eGFR* estimated glomerular filtration rate, *LVEF* left ventricular ejection fraction, Echocardiography was performed on day 2 in STEMI patients

*P < 0.05 vs. healthy controls; †*P* < 0.05 vs. stable CHD controls; ^#^*P* < 0.05 STEMI MACE (+) vs. STEMI MACE (−)

All STEMI patients were followed-up for 3 years after STEMI onset. The occurrence of a first major adverse cardiovascular event (MACE) was regarded as the follow-up endpoint, which included cardiovascular death, non-fatal ischemic stroke, recurrent MI, need for emergency or repeat revascularization, and re-hospitalization for heart failure, as defined previously [24]. Thirty-three patients suffered from MACEs were recorded, including seven cardiovascular deaths, three non-fatal ischemic strokes, one recurrent MI, 12 emergency or elective repeat revascularization, and ten readmissions for heart failure (Table 1). When comparing STEMI patients suffered MACEs (MACE[+]) to those with event-free survival (MACE[−]), MACE (+) patients showed lower LVEF and higher SYNTAX score (Table 1). All STEMI patients presented a higher increase of LPS and d-lactate on day 2, and a significant difference was observed between MACE (+) and MACE (−) patients in plasma d-lactate on the day 2 of STEMI onset.

### Gut-associated bacteria are enriched in systemic microbiome

**Fig. 1:**
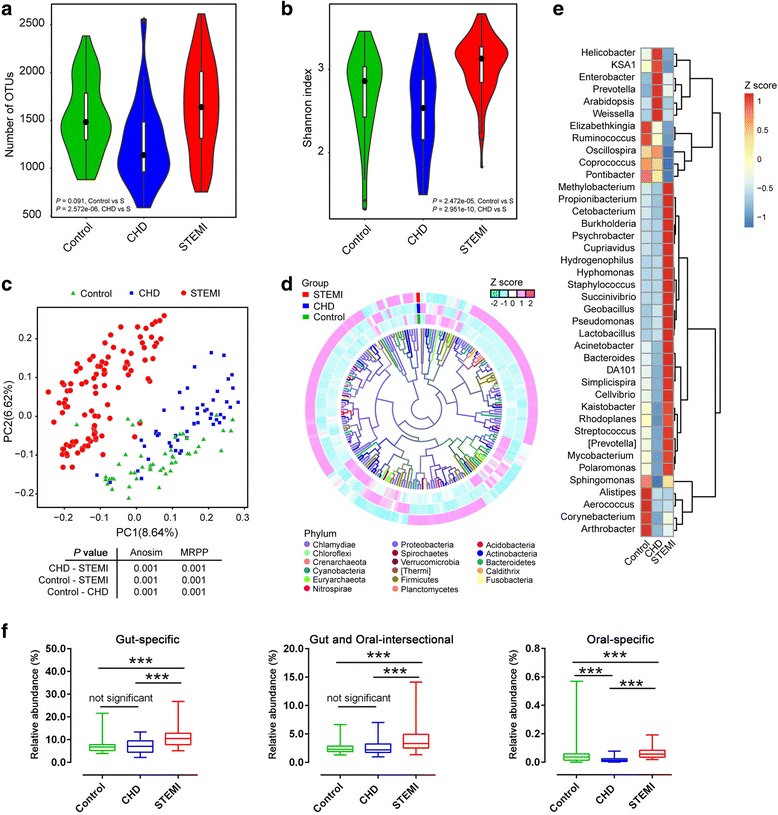
Elevated richness of microbiota in the blood of in human adults with STEMI. **a–b**The microbial richness and *α*-diversity (as accessed by Shannon index) based on the genera profile in control (*n* = 49), CHD (*n* = 50), and STEMI patients (*n* = 100). The distribution and density of samples are displayed in violin plots. Boxes represent the interquartile ranges, the inside black plots represent the median, and circles are outliers. *P* values are from Wilcoxon rank sum test. **c** Principal coordinate analysis (PCoA) based on the OTU table separate STEMI group from the controls and CHDs. Significant *P* values of Anosim and multi-response permutation procedure (MRPP) between groups emphasize the differences in microbial community structure. **d** Heatmap tree shows genera significantly different in STEMIs as compared to those in controls and CHDs, and their phylogenic relationships. The abundance profiles are expressed by *z*-scores, and genera were clustered based on Bray Curtis distance in the clustering tree. **e** Relative abundance of the top 40 most different genera across groups at adjusting *P* value ≤ 0.05 by Wilcoxon rank sum test and Benjamin and Hochberg method. The abundance profiles are transformed into *z*-scores by subtracting the average abundance and dividing the standard deviation of all samples. *z*-score is negative when the row abundance is lower than the mean. **f** The percentage of bacteria in control, CHD, and STEMI samples originated from oral and gut. More than 7.8% controls, 7.2% CHD, and 12% STEMI are derived from gut-specific microbiome. Boxes represent the interquartile ranges, the inside line represents the median. ***P < 0.001; Wilcoxon rank-sum test

**Fig. 2:**
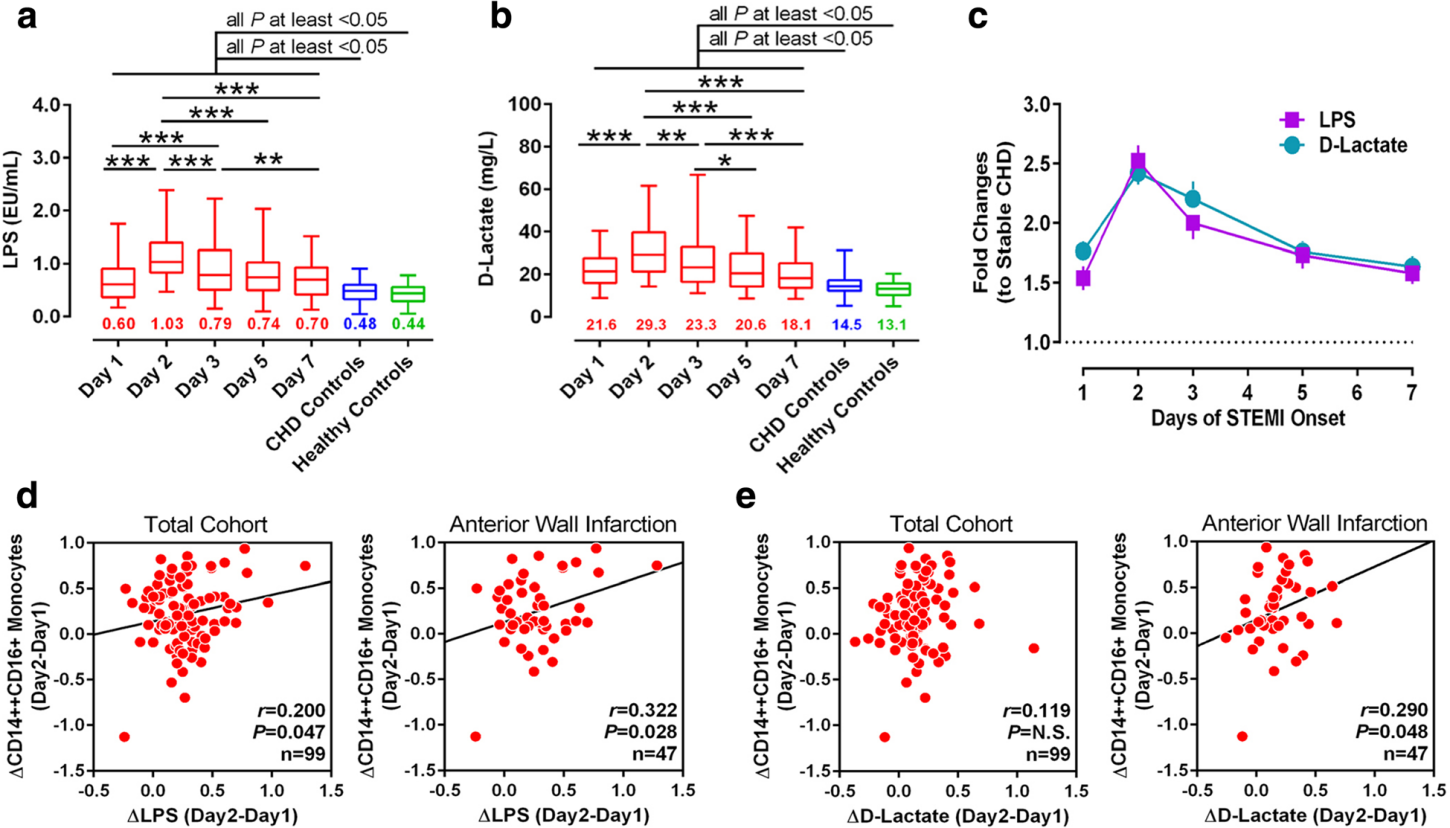
Increased plasma levels of LPS and d-lactate after STEMI are associated with CD14++CD16+ monocyte. **a–b** The dynamic changes of LPS and d-lactate in STEMI patients after admission as compared to CHD controls and healthy controls. The data are presented by box and whisker plots: the boxes extend from the 25th to the 75th percentile, with a line at the median. The whiskers extend above and below the box to show the 5th–95th percentiles of values. The number below each box and whisker plot shows the median value. *P < 0.05, **P < 0.01, ***P < 0.001, Kruskal-Wallis test followed by a Dunn’s test. **c** The temporal profiles of LPS and d-lactate levels in STEMI patients as fold changes to stable CHD controls. Data are presented as mean ± s.e.m. **d–e** The association of plasma Δ LPS and Δ d-lactate (all Δ are calculated after logarithmic transformations) with CD14++CD16+ monocytes. The left panels show the result from all STEMI patients, and right panels show the subgroups of anterior MI patients. Correlation coefficients are reported as Pearson linear correlations

As the product for gut bacterial translocation (LPS and d-lactate) in STEMI patients was significantly increased and peaked on day 2 of symptom onset, the blood samples drew on day 2 was used for microbiota analysis (Fig. 1 and Fig. 2a-c). The bacterial 16S rDNA in plasma samples was extracted and sequenced on the Illumina platform. A total of 10,560,437 high-quality sequences were generated, with an average of 53,336 per sample (Additional file 1: Table S1). The total number of operational taxonomic units (OTUs) was higher in STEMI patients than that of controls and CHDs (Fig. 1a). Consistently, the within-sample (*α*) diversity (present as Shannon index) of STEMI group was also increased (Fig. 1b). The elevated OTUs and bacterial diversity indicate more abundant bacteria in the systemic circulation of STEMI patients. Principal Coordinate Analysis (PCoA) (unweighted UniFrac distance metric based on the OTU table) showed that blood bacterial characteristics in STEMI patients were clearly separated from that in either control or CHD group (all *P* < 0.001 by Anosim analysis and multi-response permutation procedure (MRPP)), although CHD group was also distinct in comparison with healthy controls (Fig. 1c). To identify the taxonomic information of the blood bacteria, the RDP classifier and GreenGene database were used. At the phylum level, the blood dominant bacteria were from *Proteobacteria*, *Cyanobacteria*, *Firmicutes*, *Bacteroidetes*, *Actinobacteria* and *Fusobacteria*, *Euryarchaeota*, *Acidobacteria*, *Nitrospirae*, and *Chloroflexi* (Additional file 2: Table S2, Additional file 3: Figure S1), similar to the previous report [27]. In STEMI patients, a total of 210 genera were at significant abundance differences, and the genera abundance distribution was quite discrepant to that of controls and CHDs (Fig. 1d, Additional file 2: Table S3). The heat map (according to top 40 the most different genera) showed the specific genera distribution among each group (Fig. 1e). Based on reference genomes in HMP database from the human gut and oral, we found more than 12% of post-STEMI plasma bacteria were dominated by intestinal-sourced-flora, distinguishing from CHD and control group (Fig. 1f). The possibility was raised that intestinal bacteria might invade into blood post-MI, leading to the increased richness and distinct structure of systemic microbiome.

### Products of gut bacterial translocation are associated with inflammation and LV function

LPS and d-lactate were recognized as products and systemic markers for increased gut permeability and bacterial translocation [28, 29]. To measure LPS and d-lactate, blood samples of STEMI patients were collected on days 1, 2, 3, 5, and 7 of symptom onset. And the dynamic changes of LPS and d-lactate in STEMI patients were obtained. In comparison to control and CHD patients, STEMI patients’ plasma LPS and d-lactate levels increased since admission, reaching the peak on day 2 (Fig. 2a–c) and maintaining the tendency to day 7. Interestingly, the changes of LPS and d-lactate were consistent with monocytosis [24]. A prominent feature for the post-infarction inflammation is the recruitment of monocyte’s subpopulations [30–33], and CD14++CD16+ monocytes represent unique pro-inflammatory properties associated with MI [34]. To investigate the possible correlation between gut bacterial translocation and monocytosis, the association of LPS, d-lactate (Δ-value of days 2–1) with monocyte counts was analyzed. Increased plasma LPS positively correlated with total monocytes, CD14++CD16– counts (Additional file 4: Figure S2a–b) and CD14++CD16+ subset’s recruitment in the total cohort (*r* = 0.214, *P* = 0.033), especially in anterior wall infarction patients (*r* = 0.388, *P* = 0.007; Fig. 2d). However, the correlation between d-lactate and monocyte subsets was not striking (Fig. 2e, Additional file 4: Figure S2c–d**)**. Intriguingly, although d-lactate and LPS in STEMI patients have the same trend during the 7 days of observation, changes of d-lactate but not LPS were negatively correlated with LVEF by analyzing the correlation coefficients (Additional file 5: Figure S3). The association of Δ LPS and LVEF did not reach statistical significance. These findings indicated that inflammatory response after MI was partly associated with gut bacterial translocation.

### Gut microbial translocation promotes cardiovascular events after STEMI

**Fig. 3:**
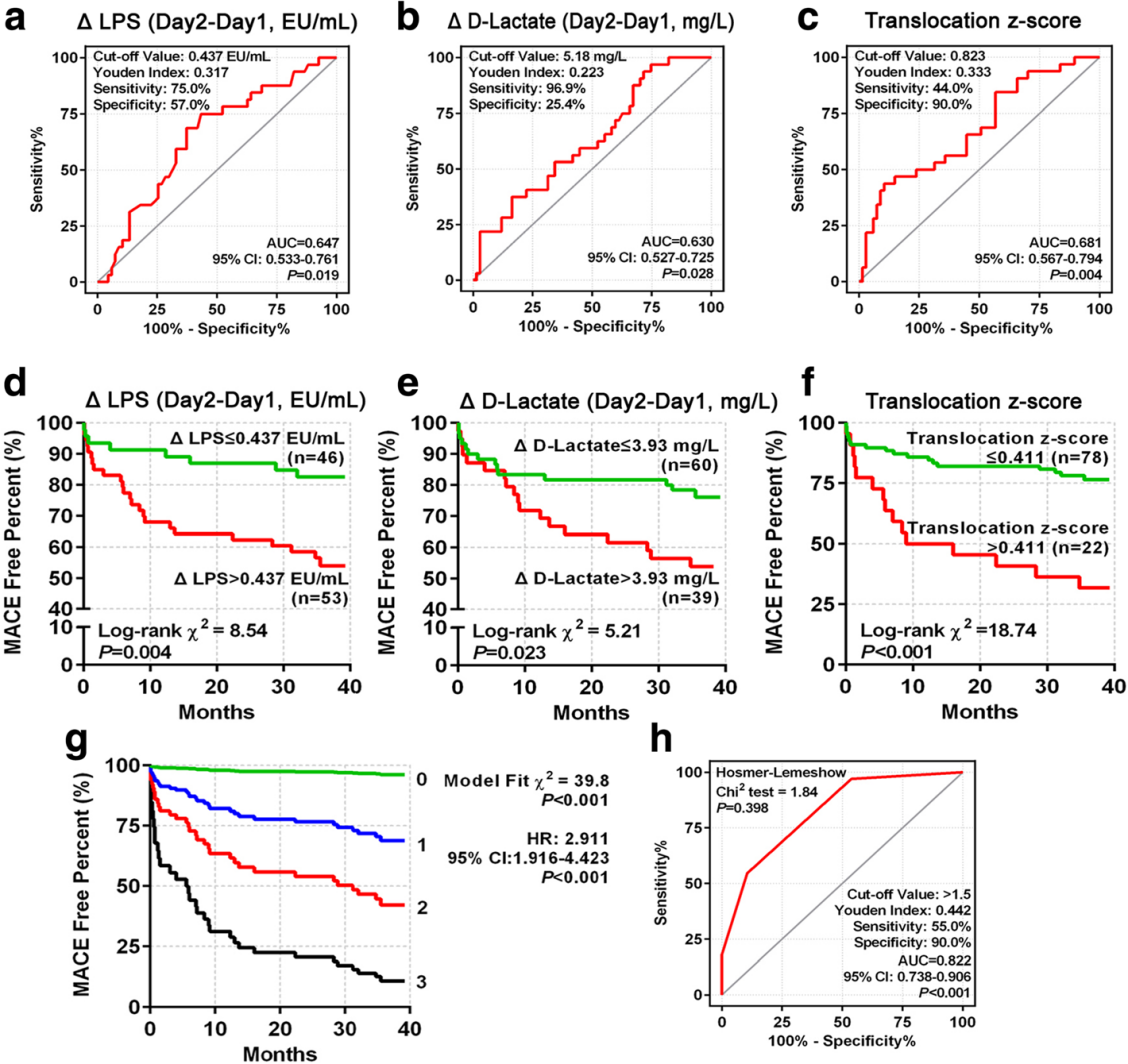
Higher LPS and d-lactate post-STEMI predict a poorer cardiovascular outcome, and TCM score was established. **a–c** ROC curve analysis of Δ LPS, Δ d-lactate, and translocation *z*-score for 3-year MACEs. **d–f** The univariate Kaplan-Meier survival analysis of Δ LPS (days 2–1), Δ d-lactate (days 2–1) and translocation *z*-score for 3-year MACEs. The patients are stratified according to the optimal cut-off values derived from ROC curve analyses. **g** The multivariate-adjusted Cox regression survival plot (with adjustment of age, LVEF (day 2) and SYNTAX score as categorical variables). The number adjacent to each line indicates the TCM score value. **h** The ROC curve analysis of TCM score

The elevated monocytosis was associated with cardiovascular events post-STEMI [24]. Therefore, we assessed the possible role of gut microbial translocation in cardiovascular events. The discriminative and prognostic capacities of Δ LPS and Δ d-lactate were confirmed in receiver operator characteristic (ROC) curve analysis (Fig. 3a, b), the performance of which was higher than other day values (Additional file 6: Figure S4). Kaplan-Meier analyses further verified that the optimal cut-off values for Δ LPS (Fig. 3d) and Δ d-lactate (Fig. 3e) were capable of identifying subjects with higher risk for post-STEMI MACEs. To incorporate the influence of the two translocation markers, we calculated the *z*-score values of Δ LPS and Δ d-lactate, and defined the sum of two *z*-score values divided by 2 as translocation *z*-score. Kaplan-Meier survival plot and ROC curve analysis showed that translocation *z*-score led to a more efficient risk stratification, increased Youden index, and improved AUC of ROC curve (*P* for multiple ROC comparisons, 0.080; Fig. 3c, f). By performing a forward stepwise selection, an optimal Cox proportional hazards model was constructed: among the candidate variables, only translocation *z*-score, hs-CRP, and CD14++CD16+ monocytes remained statistical significances in multivariate analysis. Syntax score and LVEF were excluded from the final model. To simplify their synergistic effects, we defined a simple risk score, i.e., TCM score (using the combination of the three capital letters for three markers), based on the optimal cut-off values in ROC analysis for MACEs (Additional file 1: Table S1). The multivariate Cox regression curve showed that TCM score was efficient for risk stratification by showing clearly separated survival curves as TCM score increasing from 0 to 3, even after adjustment by age, LVEF (day 2), and syntax score (Fig. 3g). ROC curve analysis further demonstrated that TCM score had an AUC of 0.822 (95% CI 0.738–0.906, *P* < 0.001) and was well calibrated (*P* = 0.398 by Hosmer-Lemeshow’s test) for MACEs (Fig. 3h).

**Fig. 4:**
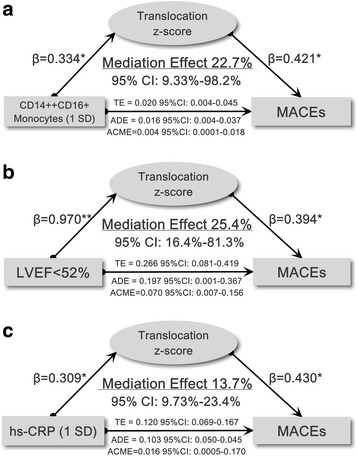
Schematic diagrams showing the coefficients relating inflammation markers, compromised LVEF with adverse cardiovascular events and the mediation effect of translocation *z*-score. **a** Association between CD14++CD16+ monocytes and MACE mediated by translocation *z*-score. **b** Association between compromised LVEF (derived from Youden index for MACE) and MACE mediated by translocation *z*-score. **c** Association between hs-CRP and MACE mediated by translocation *z*-score. β indicates coefficients in regression analysis; TE, total effect; ADE, average direct effect; ACME, average causal mediation effect; CI, confidence interval; MACE, major adverse cardiovascular events; LVEF, left ventricular ejection fraction; hs-CRP, high-sensitivity C-reactive protein. *P < 0.05, **P < 0.01

Previous studies and our recent work have demonstrated hs-CRP, CD14++CD16+ monocytes, and LVEF are predictors for post-STEMI MACEs. We next sought to examine whether and how much microbial translocation could explain or mediate such associations. By using causal mediation analysis, we demonstrated that translocation *z*-score had a significant mediation effect (22.7%, *P* < 0.05) on the CD14++CD16+ monocytes→MACEs association (per 1 SD increase), a significant mediation effect (25.4%, *P* < 0.05) on the LVEF→MACEs association (LVEF < 52%), and a significant mediation effect (13.7%, *P* < 0.05) on the hs-CRP→MACEs association (per 1 SD increase) (Fig. 4). Thus, microbial translocation could partially explain post-STEMI inflammatory response and compromised LV function-associated adverse outcomes.

### Gut permeability upon MI leads to microbial translocation and inflammation

**Fig. 5:**
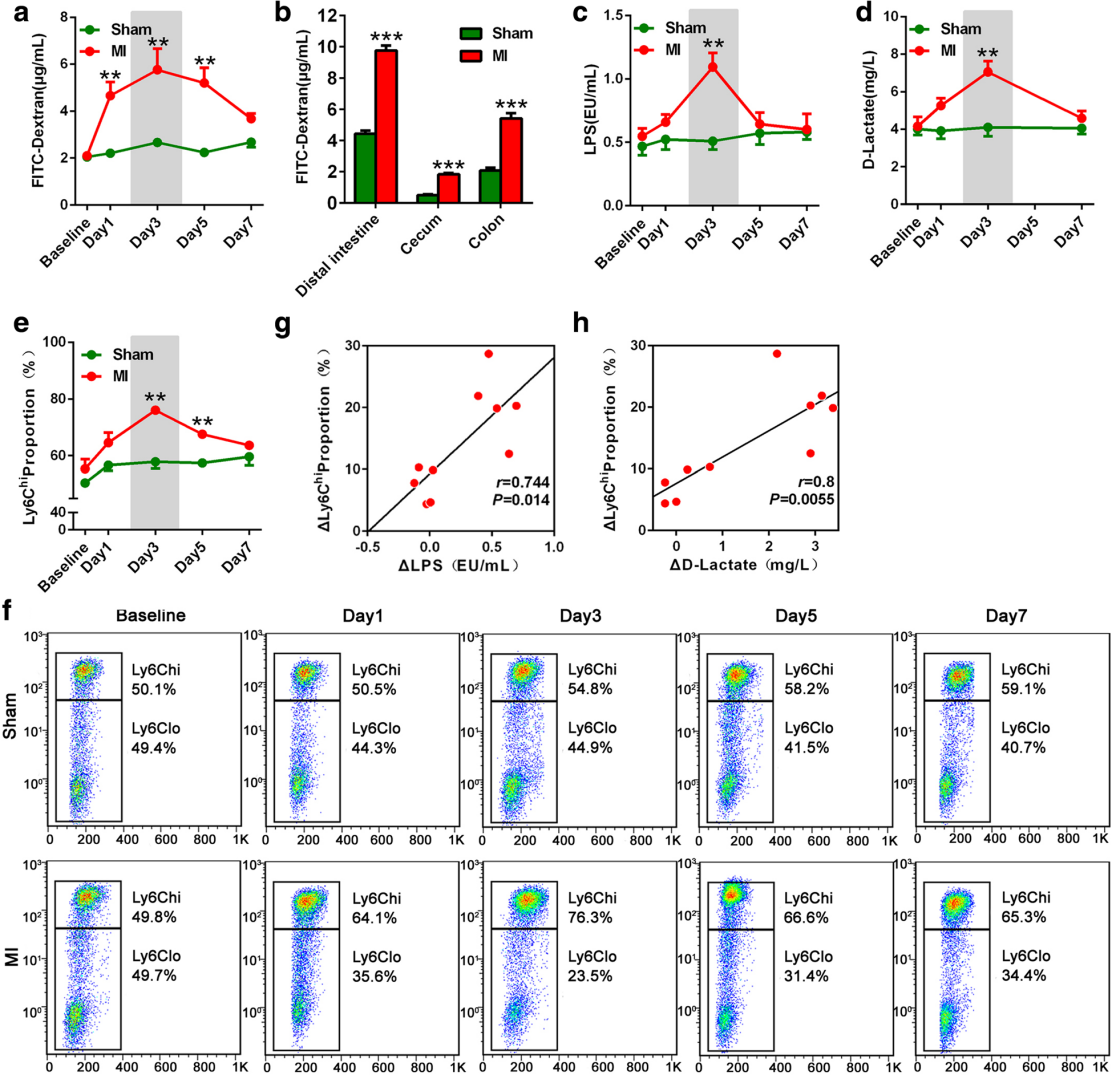
Increased gut permeability and microbial products are associated with monocyte in mice model of MI. **a** Gut permeability is elevated in the colon of MI mice. *n* = 4–6 for each group. **b** The augments of permeability in the distal small intestine, cecum, and colon at the third day of MI. *n* = 6 for each group. **c–d** The serum levels of LPS and d-lactate are enhanced after MI. *n* = 5 for each group. **e–f** The Ly6C^hi^ monocyte proportion in MI mice is significantly increased as compared to the sham group. *n* = 5 for each group. **g–h** LPS and d-lactate are positively correlated with Ly6C^hi^ monocyte proportion. *n* = 5 for each group. Correlation coefficients are reported as Pearson linear correlations. Data are presented as mean ± s.e.m. **P < 0.01; ***P < 0.001, vs respective shams, *t* test

To investigate the gut permeability contribution to microbial translocation, MI mice model was used. By fluorescein isothiocyanate (FITC)-dextran tracing assay, colon permeability increased from days 1 to 5 after MI, and recovery at day 7 (Fig. 5a). As colon, the permeability of distal small intestine and was also elevated (Fig. 5b). Accordingly, gut bacterial products (LPS and d-lactate) were increased and peaked on day 3 (All *P* < 0.01, Fig. 5c, d). In MI mice, the changes of the Ly6C^hi^ proportion monocyte subset showed the similar tendency (All *P* < 0.01, Fig. 5e, f), and the proportion of Ly6C^hi^ monocytes was positively correlated with peripheral blood concentration of LPS as well as d-lactate, consistent with STEMI patients (Fig. 5g–h). Thus, this mouse model was further used for mechanisms of gut permeability post-MI.

**Fig. 6:**
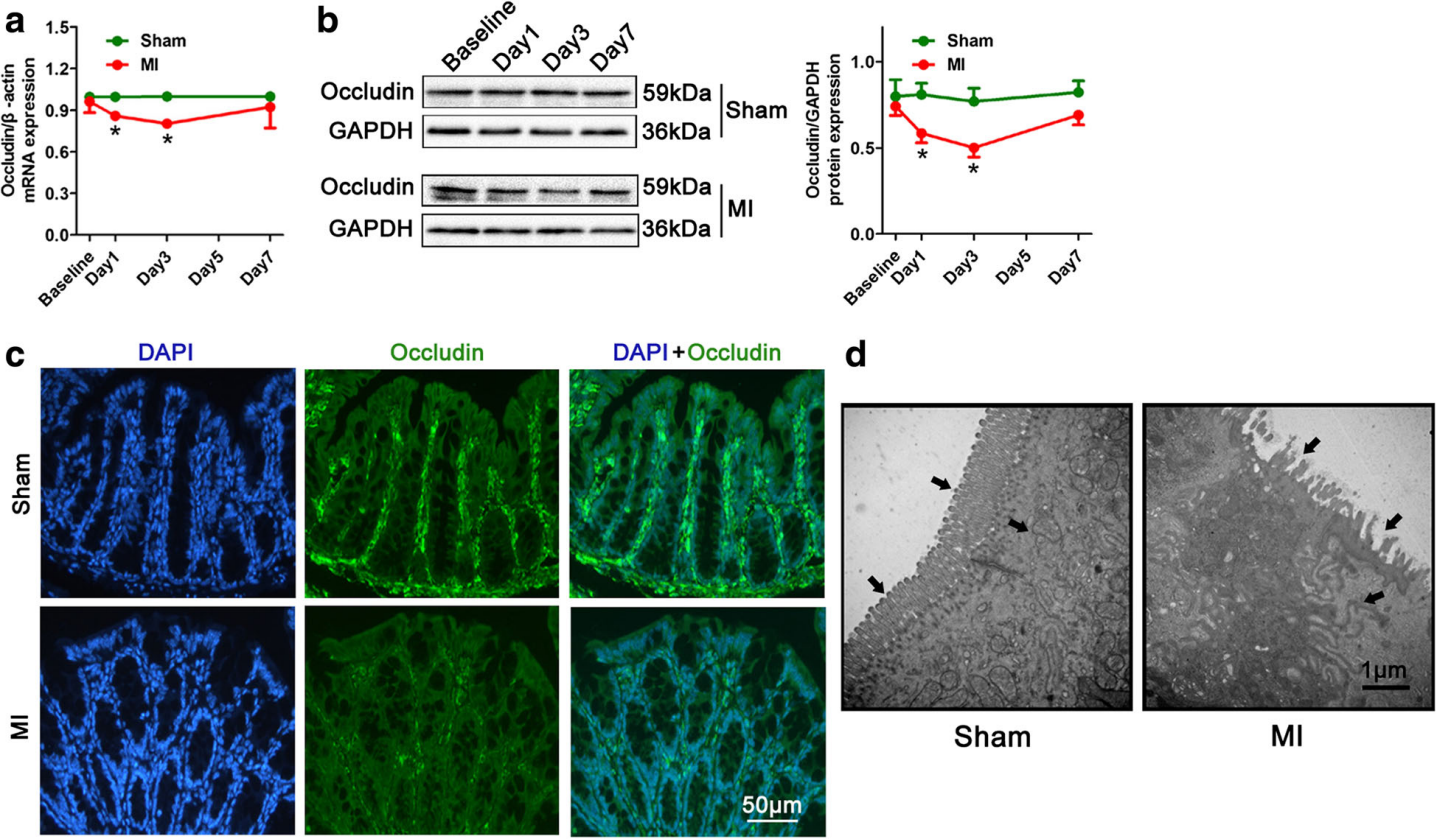
The loss expression of tight junction protein occludin post-MI leads to intestinal mucosal injury. **a** The mRNA levels of occludin in colon tissues are decreased post-infarction. *n* = 5 for each group. **b** Western blot analysis of occludin protein expression in relative to GAPDH in the colon from MI mice**.** *n* = 5 for each group. **c** Representative immunofluorescence images of occludin (green) in the intestinal epithelium of MI mice. Nuclei are stained with DAPI (blue), and scale bars are 50 μm. **d** Transmission electron microscope image of sections from the intestinal epithelium of MI mice. Scale bars are 1 μm. Data are presented as mean ± s.e.m. *P < 0.05, vs respective shams, *t* test

With gut permeability changes, occludin (tight junction protein as the intestinal permeability marker) mRNA and protein expression in the colon was dramatically decreased after MI (Fig. 6a, b). In association with occludin expression downregulation in the mucous membrane of intestinal epithelium (Fig. 6c), mucosal microvillus disarrangement, structure disrupted, and even loss were detected by transmission electron microscopy after MI (Fig. 6d). In keeping with these changes, blood flow of superior mesenteric artery decreased from day 1 and reached the nadir on day 3 (*P* < 0.01; Additional file 7: Figure S5a), which was negatively correlated with FITC-dextran permeating in the small intestine (Additional file 7: Figure S5b), but positively correlated with LVEF (*r* = 0.64, *P* < 0.001; Additional file 7: Figure S5c). These data suggested that elevated gut permeability was due to the intestinal mucosa barrier dysfunction by ischemia stress after MI.

### Inhibition of gut microbial translocation reduces infarct size

**Fig. 7:**
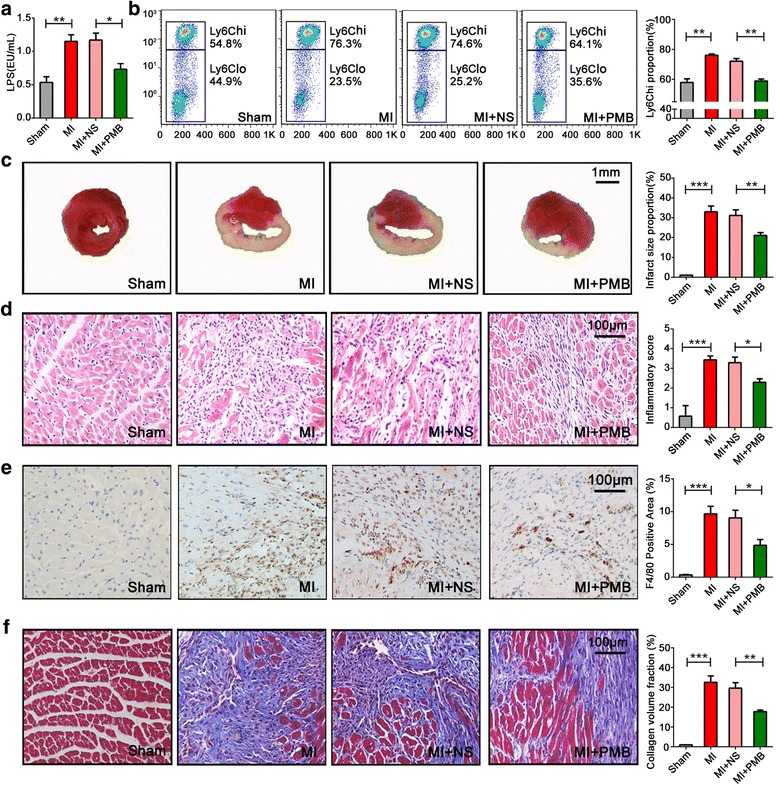
Treatment of PMB eliminate serum LPS, alleviated monocytosis, and cardiomyocyte injury post-infarction. **a** The serum levels of LPS post-MI are suppressed by administration of PMB. *n* = 3–5 for each group. **b** The Ly6C^hi^ monocyte proportions in MI mice are decreased in the presence of PMB. *n* = 5 for each group. **c** The infarct areas post-MI are reduced by PMB. *n* = 5 for each group. **d–f** Cardiomyocyte inflammation, macrophage infiltration, and collagen areas induced by MI are alleviated after PMB treatment. *n* = 5 for each group. NS, normal saline. All the data were from the third day of MI. Data are presented as mean ± s.e.m. **P < 0.01; ***P < 0.001, one-way ANOVA with Tukey’s post hoc test

To suppress gut bacterial translocation and eliminate microbial products in systemic circulation, polymyxin B (PMB; an antibiotic with distinct cationic characteristics against LPS-induced toxicity) was used to treat MI mice. By PMB administration for 3 days, the plasma LPS level was significantly reduced (Fig. 7a), and the proportion of Ly6C^hi^ was coincidently decreased (Fig. 7b). At other time points post-infarction, PMB treatment also lowered the Ly6C^hi^ counts although LPS was no changed (Additional file 8: Figure S6).

Consistent with decreased LPS and Ly6C^hi^, PMB treatment significantly lowered infract size, as determined by triphenyltetrazolium chloride stainings (Fig. 7c). Accordingly, PMB treatment also reduced local inflammation (Fig. 7d), macrophage infiltration (Fig. 7e), and myocardial fibrosis (blue area, by Masson stainings; Fig. 7f). These findings confirmed that by abrogation of gut bacterial translocation, inflammatory response and heart injury post-MI could be improved.

## Discussion

**Fig. 8:**
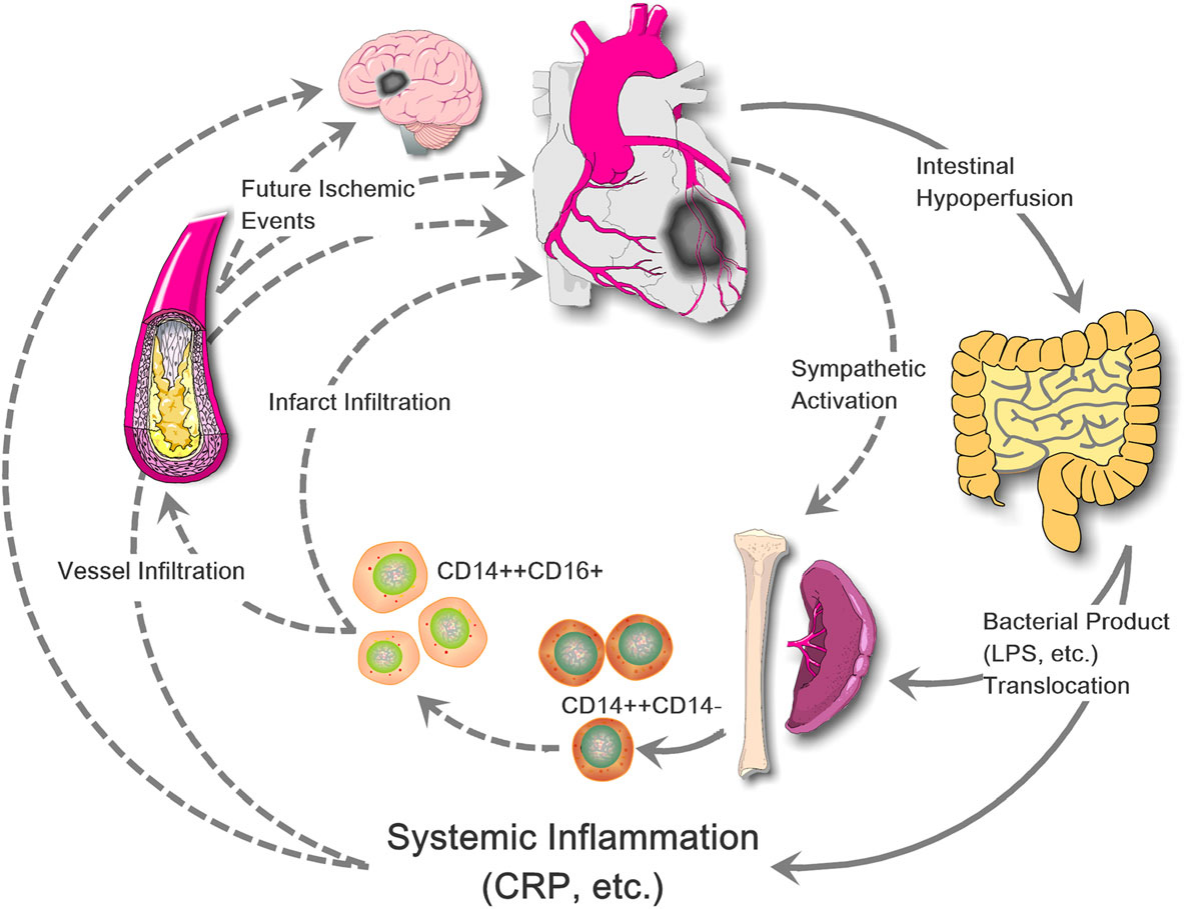
Schematic illustration of the proposed interaction between STEMI, microbial translocation, and future ischemic events. Increased gut permeability and bacterial translocation from the gut lumen due to intestinal hypoperfusion post-STEMI are involved in the pathogenesis of monocytosis and inflammation and have the potential of contributing to further ischemic events and heart failure

The present study provided convincing evidence that increased gut bacterial translocation into systemic circulation due to intestinal hypoperfusion and gut permeability post-MI contribute to the pathogenesis of future cardiovascular events by inducing inflammation (Fig. 8). New therapeutic intervention by antibiotics targeting gut bacteria and protecting gut function may be a potential option to improve cardiovascular outcomes post-infarction. This notion is derived from our novel findings that (1) increased gut microbial components and products translocation into the systemic circulation conduce to inflammation post-MI, (2) the increase of gut bacterial translocation products has great prognostic significance for cardiovascular outcomes, (3) the loss of occludin and intestinal mucosal injury is responsible for elevated gut permeability and microbial translocation post-infarction, and (4) cardiomyocyte injury post-infarction could be alleviated dramatically by gut microbial translocation inhibition.

There are approximately 10^12^ flora bacteria colonized in the human gastrointestinal tract, with the opportunistic pathogens at 10^9^ [13]. However, blood microbiome is seldom investigated as systemic circulation was previously presumed to be sterile, and circulating bacteria was thought to be in sepsis cases. Nevertheless, the bacterial DNA in blood samples is detectable, although quite low [35–37]. In blood microbiome of STEMI patients, we detected *Lactobacillus*, *Bacteroides*, and *Streptococcus*, which were probably from the intestinal flora. Moreover, the great bacteria richness and distinct microbial community drove us to explore the origin of bacteria. It was interesting that the STEMI-enriched bacteria were dominated by intestinal-sourced flora. For instance, *Bacteroides*, which has been detected in gut samples [37], was significantly more abundant in STEMI patients. The intestinal flora might be possibly the most important factor that results in enhanced microbial richness and diversity observed in STEMI patients.

The translocation of gut-originated bacteria into systemic circulation was most likely due to intestinal mucosal injury and gut barrier failure. As previously reported, the abundance of microbiota is a major difference in the luminal environment between the small intestine and colon, with the small intestine harboring ~ 10^3^ bacteria and the colon harboring ~ 10^11^ bacteria per gram of intestinal contents [38]. Despite the fact that we observed an almost ~ 1-fold increase of FTIC-dextran in the small intestine compared with that in the colon, in terms of bacterial translocation, the impact this difference is minimal considering huge difference in bacterial abundance. Additionally, a previous report showing that the colon is more efficient at eliminating translocating bacteria and presents with higher trans-epithelial resistance than the small intestine [39]. Therefore, we investigated the expression of occluding in the colon and found the loss of occludin attributed to elevated gut permeability and microbial translocation post-infarction.

Furthermore, in patients with stable heart failure, suppressed blood flow in intestinal arteries leads to bacterial products translocation into the systemic circulation [40]. Thus, we wondered whether gut barrier failure was associated with mesenteric artery blood flow in patients suffering MI. We found that d-lactate, a marker of gut barrier loss and increased gut permeability [28, 29], was inversely correlated with LVEF, which was positively linked to mesenteric artery blood flow. Experimental evidence also confirmed the negative association between increased gut permeability and mesenteric artery blood flow. Thus, the intestinal mucosal injury and gut barrier dysfunction post-MI was probably driven by ischemia stress such as compromised LV function and intestinal hypoperfusion.

To investigate the possible link between bacterial translocation and systemic inflammation, we used hs-CRP as a predictor for MACEs and found that hs-CRP had a very strong impact on adverse outcomes. Moreover, hs-CRP, CD14++CD16+ monocytes, and translocation *z*-score were the only predictors with remained statistical significances in the final Cox model. To simplify their clinical significance, we defined TCM score to integrate their synergistic effects, which presented with an improvement in risk stratification compared with any predictor alone. By using causal mediation analyses, translocation *z*-score could partially explain CD14++CD16+ monocytes and hs-CRP-associated MACEs, further supporting microbial translocation as an underlying triggering mechanism for systemic inflammatory response after STEMI, where the magnitude of which poses the risk for future adverse cardiovascular outcomes.

Myocardial depression is a common feature of LPS-induced endotoxemia [41, 42]. The plasma levels of LPS were reported to be slightly elevated in acute MI patients with left ventricular dysfunction [15]. But there was no statistical significance and the sample size was quite small. We enrolled 100 STEMI patients and confirmed that LPS was elevated as a result of gut microbial translocation. Therefore, it was speculated that the accumulation of gut bacteria and intestine-produced LPS in systemic circulation triggers monocyte’s recruitment, which activates systemic inflammation and eventually heart injury. This is in agreement with the findings that CD14++CD16+ monocytes act as an initiator of early immune response to local microbial infections [43]. By stimulation with bacterial products like LPS, the monocyte subpopulation had been shown to efficiently produce the pro-inflammatory cytokine TNF and subsequently regulate the immune response [44]. This gut-driven concept is also supported by a report showing that intestinal bacteria could be direct innate immune cell development directly via promoting hematopoiesis [45]. Recent evidence that the human bone marrow harbors a CD14++CD16+ monocyte pool [46] raise the possibility that the recruitment of circulating CD14++CD16+ monocytes after STEMI may be directly mobilized from the bone marrow or spleen reservoir. It is possible that the gut bacterial components and LPS-induced monocytosis and systemic inflammation observed in our study is mediated by monocyte’s mobilization on the bone marrow or spleen reservoir [47]. However, the contribution of gut-induced hematopoiesis vs. mobilization remains to be investigated.

In recent clinical trials, increasing efforts have been devoted to reducing the cardiovascular events of MI patients by antibiotic administration. MI patients treated with antibiotics exhibit a reduction of 36% in all endpoints during the 1-year follow-up in the STMINA trial [48]. According to the newest findings of the TIPTOP trial, a timely administration of doxycycline reduces the adverse LV remodeling in patients with acute STEMI and LV dysfunction [49]. However, the underlying mechanism was not identified yet. The novel findings in the current study that gut-dependent microbial translocation induces cardiovascular events after MI provided insight into the mechanism of antibiotic treatment in improving cardiovascular outcomes. Therefore, suppression of gut microbial translocation by antibiotics would be an optimal approach for reducing post-infarction injury and promoting cardiac repair.

Here, this study indicated that gut-dependent microbial translocation induces myocardial inflammation and injury. We have proven that inhibition of gut microbial translocation could reduce local inflammation, suppress monocyte infiltration, and reduce infarct size. However, whether white blood cells originating from the gut directly translocate into the myocardium and contribute to infarction is unknown and warrants future investigation.

## Conclusions

Taken together, our data demonstrate for the first time that MI-induced acute LV dysfunction and intestinal hypoperfusion cause intestinal mucosa injury and gut barrier failure, trigger gut microbial and product’s translocation into systemic circulation, mobilize monocytes, and initiate the inflammation, which consequently predisposes patients to cardiovascular events. This work has revealed the crucial contribution of gut microbial translocation in inflammation and cardiovascular events after MI and highlighted the therapeutic potential of gut-targeted approaches to reduce the incidence of cardiovascular events post-MI.

## Methods

### Study cohort and patient characteristics

The patients admitted to Pingjin Hospital Heart Center from November 2012 to May 2013 in our previous work were enrolled [24]. The diagnosis and treatment of STEMI were performed according to the guideline recently published [50]. Patients with infectious and inflammatory disorders, cancer, previous MI, and decompensated heart failure in the past 6 months were excluded. Individuals were also excluded if they had received antibiotics, probiotics, or hormone-replacement therapy within the last 8 weeks. All the STEMI patients have taken aspirin prior to primary PCI. PCI was performed with conventional techniques and coronary stents were used without restrictions. The infarct-related artery was the only target of the procedure. Finally, there were 49 healthy controls, 50 stable CHDs, and 100 STEMI patients in the study cohort. The admission blood samples were collected after initial diagnosis and prior to the first antiplatelet medication. The study was performed in accordance with the Helsinki declaration and was approved by the hospital Research Ethics Committee. Informed consent was obtained from study participants.

### Blood biochemical testing

Baseline blood routine test and biochemical assays were performed at admission using an automated hematology analyzer (XE-5000, Sysmex, Kobe, Japan) and a Hitachi 7180 Clinical Analyzer (Hitachi, Tokyo, Japan). High-sensitivity C-reactive protein on day 2 of STEMI onset was assayed by a commercially available ELISA kit (CUSABIO, Wuhan, China; Cat No. CSB-E08617h) according the manufacturer’s instruction.

### LPS and d-lactate measurement

Fasting blood samples were collected from subjects in control, CHD, and STEMI group via the antecubital vein in ethylenediaminetetraacetic acid anti-coagulated tubes. The whole blood samples were centrifuged at 3500 rpm for 10 min at 4 °C and separated into plasma. Plasma samples were subsequently used for LPS and d-lactate measurement. The levels of LPS were determined by a kinetic chromogenicmethod-based Tachypleusamebocyte lysate assay (China Horseshoe Crab Reagent Manufactory Inc., Xiamen, China; Cat No.KT22) according to the manufacturer’s instruction. The kit provides pretreatment reagent that eliminated the inhibition factors in plasma. The assay range of the test was 0.01–10 EU/ml, and the precision was 0.005 EU/ml. d-lactate levels were measured using a d-lactic acid (d-lactate) (Rapid) assay kit (Megazyme, Bray, Ireland) as the others did previously [51]. The absorbance of the sample was detected at 340 nm in a spectrophotometer. The assay range of the test was > 0.214 mg/L. All the samples were assayed in duplicate.

### Follow-up

All patients were followed-up after STEMI onset as we described previously [24]. To date, all the patients have finished a follow-up for 3 years since STEMI onset. Thirty-three first major adverse cardiovascular events were detected, including seven cardiovascular deaths, three non-fatal ischemic strokes, one recurrent MI, 12 emergency or elective repeat revascularization, and ten readmissions for heart failure. The occurrence of a first MACE was regarded as the endpoint. Medical records were obtained from the treating physicians to verify all events reported by study participants (Fig. 8).

### Calculation SYNTAX score

Coronary lesion severity, as assessed by SYNTAX score, was calculated using the SYNTAX score algorithm (www.syntaxscore.com).

### DNA extraction and Illumina sequencing

Isolating genomic DNA from whole blood is classically done using the buffy coat. Bacterial DNA is extracted from peripheral blood leucocytes. Genomic DNA from 200 ul blood samples was isolated using the MoBioPowersoil DNA Isolation Kit (Mo Bio Laboratories, Carlsbad, CA, USA) following the manufacturer’s protocol. The concentration and quality of extracted DNA were assessed photometrically using a NanoDrop® ND-2000c UV–vis spectrophotometer (NanoDrop Technologies, Wilmington, DE, USA). The universal primer set 515F (5′-GTG CCA GCM GCC GCG GTA A-3′) and 806R (5′-GGA CTA CNN GGG TAT CTA AT-3′) was used for the amplification of the V4 region of bacterial 16S rRNA gene. After 16S rDNA library preparation and generation, the library quality was assessed on the Qubit@ 2.0 Fluorometer (Thermo Scientific) and Agilent Bioanalyzer 2100 system. Then, the libraries were sequenced on IlluminaHiSeq platform 2500 and 250 bp paired-end reads were generated at Novogene (Beijing, China).

### Quality filtering, OTUs picking, and annotation

Based on samples’ unique barcode, raw reads were assigned to different samples, then the assigned paired-end reads of each sample were merged to raw tags by using FLASH (Version 1.2.7) [52]. The merged raw tags were filtered and developed into clean tags according to QIIME (Version 1.7.0) quality controlled process [53]. After the quality control, clean tags were aligned to Gold database (Release 20110519) and chimera sequence was detected by using UCHIME Algorithm (Version 7.0.1001) [54, 55], these non-chimera clean tags were defined as effective tags. The effective tags were clustered into OTUs with ≥ 97% similarity by Uparse (Version 7.0.1001) [56]. The representative sequence for each OTU was selected and the taxonomic information was annotated using RDP classifier (Version 2.2) [57] and GreenGene database (Release gg_13_8) [58] (Additional file 9: Supplementary methods**)**.

### Echocardiography

Transthoracic echocardiography was performed on a Philips iE33 system (Phillips, Andover, MA, USA) at 24 h after the onset of STEMI patients. For mice model, blood flow velocity in the superior mesenteric artery (SMA) was measured using the using the Vevo 2100 High-Resolution Ultrasound System with a 40-MHz transducer (VisualSonics, Toronto, Ontario, Canada) under anesthesia with isoflurane (0.25 to 0.50%) supplemented with 100% O_2_, by an experienced sonographer. Analysis of flow velocity and sound was performed in pulse-waved Doppler mode. The angle between the Doppler beam and the mesenteric artery was < 60°. The average velocity of blood flow was determined by multiplying velocity time integral by heart rate.

### Animal model

C57BL/6J male mice at 6-week-old were randomly divided into groups of MI and sham group after adaptively feeding for 1 week, with five time points (1, 3, 5, 7 days; *n* = 5 for each group). Surgical MI was induced by the ligation of the left anterior descending coronary artery. Sham-procedure mice underwent the same protocol, but without ligation of the coronary artery. For MI+saline (NS) and MI+PMB group, normal saline was given as vehicle control, and an equal volume of PMB (2.5 μg/g) was administered by intraperitoneal injection immediately after MI induction, and on days 2 to 4 after surgery, once daily. When sacrificed, the peripheral blood was collected for measurements of LPS, and monocyte counts, heart, and colon tissues were obtained for the further study.

### Flow cytometry analysis of circulating monocyte subsets

Flow cytometry (FCM) analysis was performed using a Cytomics FC500 cytometer (Beckman-Coulter, Miami, FL) according to previous work [59]. Fifty microliters of EDTA-anticoagulated whole blood was stained with antibody mix containing 10 μL PerCP-Cy5.5 (clone 1A8), 10 μL FITC-Ly6C (clone HK1.4), and 10 μL PE-CD11b antibodies, incubated for 15 min at room temperature in the dark. The following isotype controls were used: PerCP/Cy5.5 Rat IgG2a, FITC Rat IgG2c, and PE Rat IgG2b. All antibodies were obtained from BioLegend (San Diego, CA, USA). Then, 1 mL red blood cell lysis buffer (Biolegend Red Blood Cell Lysis Buffer) was added and incubated for 10 min. Followed by centrifuge at 350*g* for 5 min, the supernatant was carefully aspirated without disturbing the cell pellet and the pellet was re-suspended using BioLegend Cell Staining Buffer. Unstained, single stained, and Fluorescence Minus One (FMO) controls were used for setting compensation and gating boundaries. The data analysis was performed using FlowJo software (Treestar, Ashland, OR, USA).

### Fluorescein isothiocyanate-dextran assessment

At 4 h before euthanasia, the mice received fluorescein isothiocyanate (FITC)-dextran diluted in water (60 mg/100 g body weight) by oral administration. An intestinal loop model was used to assess the intestinal permeability of each gastrointestinal segment [60]. Briefly, after a midline laparotomy incision, the segments of the gastrointestinal tract, including the intestine, cecum, and colon, were created with two vascular hemoclips. The length of intestine between the two clips was injected with 50 μl FITC-dextran. After 1 h, plasma was collected immediately after sacrifice, and FITC-dextran measurement was performed on an LS55 fluorescence spectrophotometer (PekinElmer Life Sciences, Cambridge, UK), with excitation wavelength at 480 nm and emission at 520 nm.

### Histology

To avoid a weaker scar and greater propensity for rupture early after MI, heart tissues of mice in the MI, sham MI+NS, and MI+PMB groups were harvested at 14 days of MI. Triphenyltetrazolium chloride staining was performed, and the entire heart based on apex sections was all assessed for infarct size. Heart tissues fixed with 4% paraformaldehyde were embedded in paraffin and sectioned. Tissue sections were stained with hematoxylin-eosin to determine the severity of myocardial inflammation. As described previously [61], a myocardial inflammation score at 0 means no myocardial fibrosis, 1 means very minimal focal subepicardial interstitial fibrosis just infiltrating beneath epicardial fat, 2 represents mild subepicardial interstitial fibrosis infiltrating deeper into the subepicardial myocardium, 3 represents multifocal subepicardial interstitial fibrosis, and 4 means replacement fibrosis. For immunohistochemical staining of F4/80-positive cells, tissue sections processed through deparaffinage, rehydration and antigen plerosised, and endogenous peroxidase activity blockade, were incubated with F4/80 antibodies at 4 °C overnight. Following secondary antibodies incubation, the sections were stained with avidin-biotin complex and counterstained with hematoxylin. Collagen volume fraction at the free wall of infarct area was assessed through Masson staining. Fixed colon tissues were embedded in OCT, stained with Occludin incubated with FITC-coupled secondary antibodies, and counterstained with DAPI. The stainings were examined under a fluorescence microscope (Nikon). For electron microscopic study, the superior mesenteric arteries were fixed in 2% glutaraldehyde/osmium tetroxide, after dehydration in ethanol and embedded in spurr resin. Thin sections were obtained from a microtome and viewed with a Hitachi 7500 transmission electron microscope (Hitachi Limited, Tokyo, Japan).

### RNA isolation and real-time quantitative RT-PCR

Total RNA was extracted from colon tissues using Trizol reagent according to the manufacturer’s instructions. The concentration of RNA was quantified by a NanoDrop® ND-2000c UV–vis spectrophotometer (NanoDrop Technologies, Wilmington, DE, USA). Total RNA from each sample was reverse transcribed with the Superscript First-Stand cDNA Synthesis Kit (Invitrogen CA, USA). The quantitative RT-PCR was performed with SYBR Green I on an ABI Prism 7300 sequence detection system (Applied Biosystem, Foster City, CA, USA). *β*-actin was used as the internal control. The specific primer sequences were as follows: *Occludin*: sense: 5′-CCACCCCCATCTGACTATGC-3′, antisense: 5′-TCGCTTGCCATTCACTTTGC-3′, length 78 bp and *β*-actin: sense: 5′-CTAAGGCCAACCGTGAAAAG-3′, antisense: 5′-ACCAGAGGCATACAGGGACA-3′, length 104 bp. The relative mRNA level of each sample was analyzed and calculated using the 2^-∆∆Ct^ method.

### Western blot

Proteins from colon tissues were extracted with cold lysis buffer. Following ultrasonication and centrifugation, the supernatant was harvested. Protein concentration was determined by the Bio-Rad protein assay kit (Bio-Rad Laboratories, Inc., Berkeley, CA, USA). Equal amounts of protein were fractionated on SDS-PAGE and transferred to nitrocellulose membranes (Millipore, USA). The membranes were blocked and incubated with primary antibodies against Occludin, and GAPDH. After binding with secondary antibodies, the protein bands were detected using a ChemiDoc™ XRS gel documentation system (Bio-Rad, Hercules, CA, USA).

### Statistical analysis

Categorical data were compared with Fisher’s exact test. For comparisons between two independent groups, an unpaired Student’s *t* test or a Mann-Whitney *U* test was used. For comparison across three groups or more, one-way analysis of variance with Tukey’s post hoc analysis or a Kruskal-Wallis test followed by a Dunn’s test were performed. Correlation analyses were performed based on Spearman’s correlation. Kaplan-Meier survival analysis followed by the log-rank test was employed to estimate cumulative adverse event-free rates. To construct a new variable that integrate the impact of Δ LPS and d-lactate, we calculated their *z*-scores (standard *z*-transformation), which was calculated as: *z* = (*x*-*μ*)/*σ*, where *μ* is the mean value and σ is the standard deviation. The sum of two *z*-scores divided by 2 was defined as translocation *z*-score (mean = 0, standard deviation = 1). Receiver operating characteristic (ROC) curve was plotted to assess the accuracy and the optimal cut-off value (the best Youden Index: sensitivity + specificity − 1) for each parameter to discriminate between MACE (+) and MACE (−) patients. Parameters with an area under the curve (AUC) of *P* value ≤ 0.1 were then used for Kaplan-Meier survival analyses and Cox proportional hazards analyses. For Kaplan-Meier analysis, STEMI patients were stratified by Youden Index-derived optimal cut-off values. To construct optimal Cox proportional hazards model for MACEs, potential variables were first transformed into binary variables by using the optimal cut-off values, and then a stepwise forward selection was performed to select variables (*P* < 0.05) into multivariate model. Model calibration was performed by Hosmer-Lemeshow’s chi-squared test.

Mediation analysis models were constructed as previously prescribed [62] to assess whether and how much microbial translocation could explain the associations between inflammatory markers compromised LVEF and adverse outcomes. Linear and logistic models were used to estimate the associations between predictors and mediator and the associations between predictors, mediator, and outcome. The predictor variables were CD14++CD16+ subset measured on day 2 (continuous variable, per 1 SD increase), hs-CRP (continuous variable, day 2, per 1 SD increase), and reduced LVEF (binary variable defined by Youden Index, day 2); the mediator variable was translocation *z*-score (continuous variable); the outcome variable was MACEs (binary variable). We estimated the total effect (TE) and average direct effect (ADE) between the predictors and outcome, the average causal mediation effect (ACME) between predictors and outcome via mediator, and the percent of mediation effect, where TE equals to the sum of ACME and ADE. We used user-written command “medeff” in STATA to perform mediation analysis [63], and the results were calculated using bootstrapping with1000 iterations. The above statistical analyses were performed using STATA version 14.1 (STATA Corp., College Station, TX, USA) and GraphPad Prism version 5 (GraphPad Prism Software Inc., San Diego, CA, USA). A two-tailed *P* value < 0.05 was considered statistically significant.

QIIME software package (Version 1.7.0) was used to analyze alpha diversity and beta diversity. For alpha diversity, the Shannon index was calculated based on the genera profile of control, CHD, and STEMI patients. Then, for beta diversity, OTU table was used to generate unweighted UniFrac distance matrix, and PCoA was performed and displayed by WGCNA package, extra font package and ggplot2 package in R software (Version 2.15.3) (Additional file 9: Supplementary methods**)**. Differential abundance of genera was tested by Wilcoxon rank sum test, and *P* values were corrected for multiple testing with Benjamin and Hochberg method.

From HMP database, the reference genomes isolated from human gut and oral were used to tracking the source of the STEMI-enriched bacteria. Based on NCBI taxonomy database, we can get the reference genomes’ taxonomic and phylogenic information. Based on the phylogenic information and the genera profile, the source of the genera was defined and the proportion of different groups was calculated (Additional file 9: Supplementary methods**)**. For differential analysis, the *P* values between different groups were calculated by Wilcoxon rank sum test.

## Additional files

Additional file 1: **Table S1**. Prognostic and discriminative capacity and definition of TCM score components. (DOCX 14 kb)

Additional file 2: **Table S2.** Data production of 198 samples in three groups (control, CHD, STEMI). Table S3. Relative abundance profile at the phylum level. Table S4. Detailed information of differential genera (adjust *P* value ≤ 0.05). (XLSX 153 kb)

Additional file 3: **Figure S1.** Box plots comparing the relative abundances of top 10 most different phylum across groups. Boxes represent the interquartile ranges, lines inside the boxes denote medians, and circles are outliers. *P* value ≤ 0.05, Wilcoxon rank sum test. (PDF 281 kb)

Additional file 4: **Figure S2.** The correlation between LPS, d-lactate, and monocyte count after STEMI. (a–b) The association of plasma Δ LPS with total monocytes and CD14++CD16-subset. (c–d) Correlation analyses of plasma Δ d-lactate with total monocytes or CD14++CD16-subset. The upper panel shows the result for all STEMI patients, and the lower panel shows the subgroups of anterior MI patients. All Δ are calculated after logarithmic transformations. Correlation coefficients are reported as Pearson linear correlations. (PDF 806 kb)

Additional file 5: **Figure S3.** The association of LPS, d-lactate with left ventricular ejection fraction (LVEF) after STEMI. (a) The relationship between Δ LPS and LVEF in all STEMI patients, and the subgroups of anterior MI patients, respectively. (b) Correlation analyses of Δ d-lactate and LVEF in total cohort and anterior MI subgroup. Correlation coefficients are reported as Pearson linear correlations. (PDF 387 kb)

Additional file 6: **Figure S4**. The ROC curve of Δ LPS and Δ d-lactate for 3-year MACEs of STEMI patients. (PDF 686 kb)

Additional file 7: **Figure S5**. The mesenteric artery blood flow in MI mice is associated with gut permeability and LVEF. (a) Echocardiographic measurements show significantly depressed blood flow of superior mesenteric artery post-infarction. *n* = 5 for each group. Data are presented as mean ± s.e.m. *P < 0.05, **P < 0.01 vs respective shams, *t* test. (b) The superior mesenteric artery blood flow is negatively correlated with gut permeability in the small intestine. (c) The association of LVEF with blood flow of superior mesenteric artery. (PDF 962 kb)

Additional file 8: **Figure S6.** The impact of PMB on LPS levels and Ly6C^hi^ monocyte proportions. (a–c) PMB significantly represses the proportions of Ly6C^hi^ monocyte at days 1, 5, and 7 post-infarction. *n* = 3–5 for each group. (d–f) There is no statistical significance of LPS levels by the treatment of PMB in mice of MI. *n* = 4–5 for each group. Data are presented as mean ± s.e.m. *P < 0.05, **P < 0.01, ***P < 0.001; one-way ANOVA followed by Tukey’s post hoc test. (PDF 1260 kb)

Additional file 9: Supplementary methods. The script and code used for microbiota analysis. (PDF 41 kb)

## Abbreviations

ACME: Average causal mediation effect
ADE: Average direct effect
AUC: Area under curve
CHD: Coronary heart disease
CI: Confidence of interval
FCM: Flow cytometry
FITC: Fluorescein isothiocyanate
FMO: Fluorescence Minus One
HR: Hazard ratio
hs-CRP: High-sensitivity C-reactive protein
LV: Left ventricle
LVEF: Left ventricular ejection fraction
MACEs: Major adverse cardiovascular events
MI: Myocardial infarction
MRPP: Multi-response permutation procedure
OTUs: Operational taxonomic units
PCI: Percutaneous coronary intervention
PCoA: Principal Coordinate Analysis
PMB: Polymyxin B
ROC: Receiver operator characteristic
SMA: Superior mesenteric artery
STEMI: ST-segment elevation myocardial infarction
TE: Total effect
