# Supplementary material for: Gut-dependent microbial translocation induces inflammation and cardiovascular events after ST-elevation myocardial infarction
Source: Microbiome. 2018 Apr 3;6:66. doi: 10.1186/s40168-018-0441-4 (PMC5883284; doi:10.1186/s40168-018-0441-4)
Supplement: Supplementary file 1 — Table S1. Prognostic and discriminative capacity and definition of TCM score components. (DOCX 14 kb) [file 40168_2018_441_MOESM1_ESM.docx]

**Additional file 1: Table S1. Prognostic and discriminative capacity and definition of TCM score components.**

| Components | UnivariateHR | 95% CI | *P* Value | Area Under ROC Curve (c-statistic) | Score Value |
| --- | --- | --- | --- | --- | --- |
| **T**: Translocation z-score (Day 2) | 4.115 | 2.057-8.231 | <0.001 | 0.681 | 1: >0.411  0: ≤0.411 |
| **C**: hs-CRP (Day 2) | 4.466 | 1.842-10.829 | 0.001 | 0.716 | 1: >2.644mg/l  0: ≤2.644mg/l |
| **M**: CD14++CD16+ Monocytes (Day 2) | 3.492 | 1.221-9.988 | 0.020 | 0.688 | 1: >25.8cells/μl  0: ≤25.8 cells/μl |

CI = confidence interval; EU: endotoxin unit; HR = hazard ratio; ROC = Receiver operator characteristic. The area under ROC curve was calculated using the components as continuous variables.
