# Supplementary figures and images for: Gut-dependent microbial translocation induces inflammation and cardiovascular events after ST-elevation myocardial infarction
Source: Microbiome. 2018 Apr 3;6:66. doi: 10.1186/s40168-018-0441-4 (PMC5883284; doi:10.1186/s40168-018-0441-4)

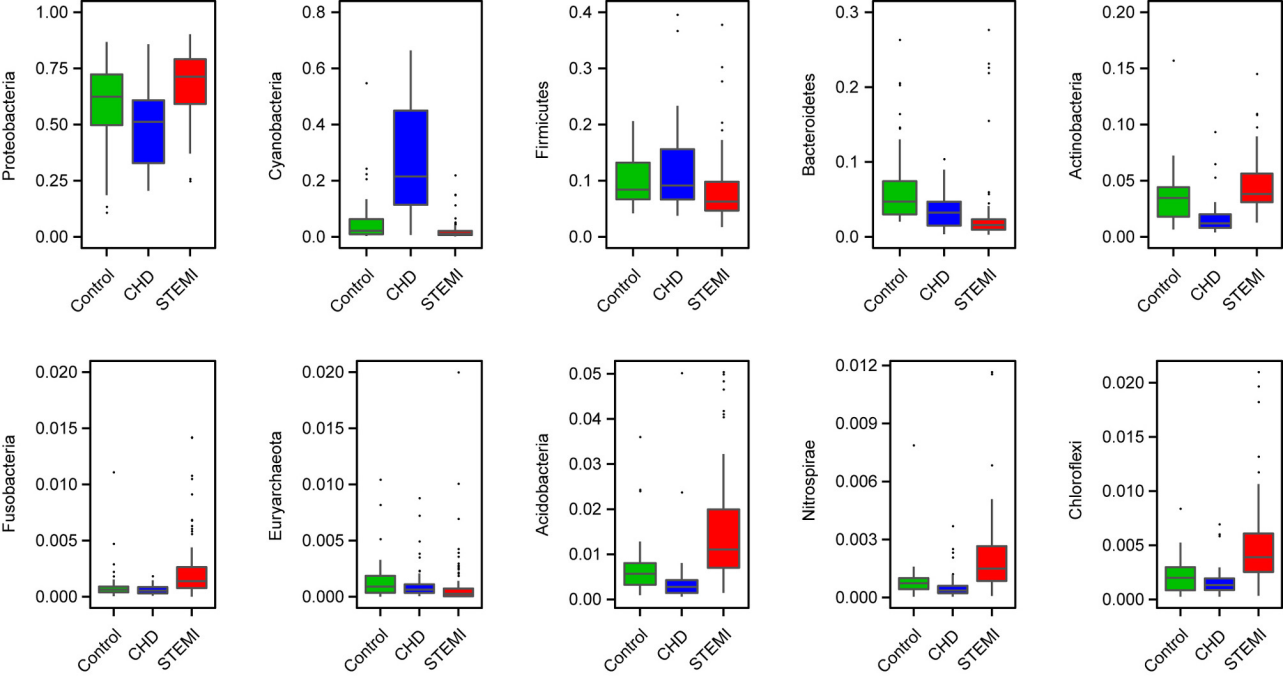

Supplement: Supplementary file 3 — Figure S1. Box plots comparing the relative abundances of top 10 most different phylum across groups. Boxes represent the interquartile ranges, lines inside the boxes denote medians, and circles are outliers. P value ≤ 0.05, Wilcoxon rank sum test. (PDF 281 kb) [file 40168_2018_441_MOESM3_ESM.pdf]

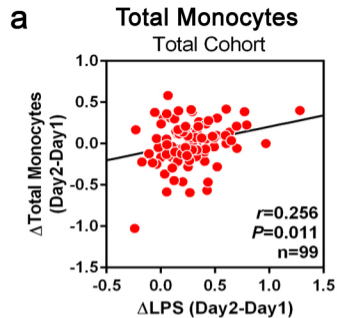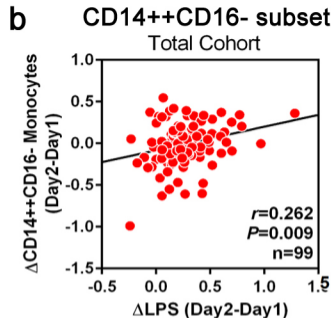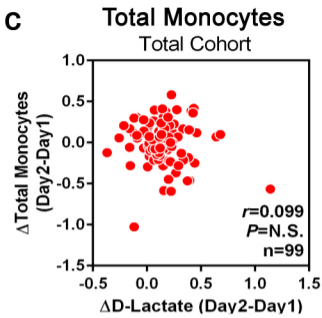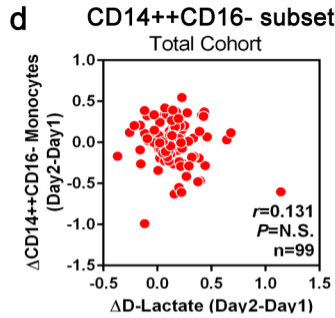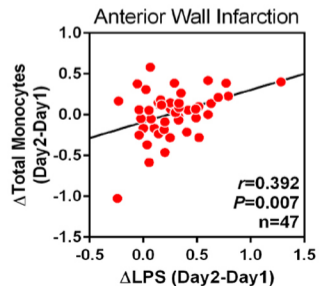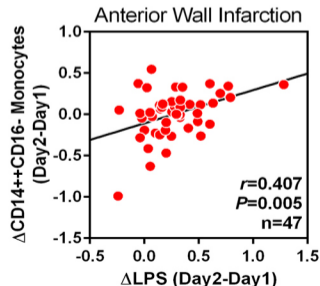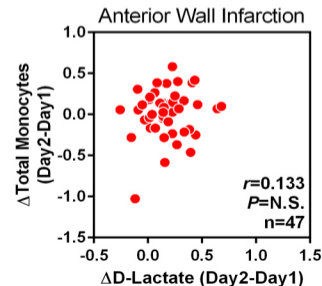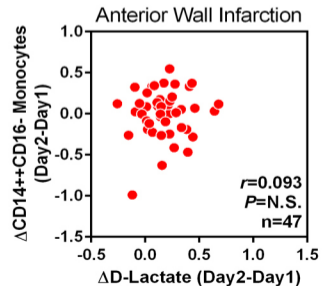

Supplement: Supplementary file 4 — Figure S2. The correlation between LPS, d-lactate, and monocyte count after STEMI. (a–b) The association of plasma Δ LPS with total monocytes and CD14++CD16-subset. (c–d) Correlation analyses of plasma Δ d-lactate with total monocytes or CD14++CD16-subset. The upper panel shows the result for all STEMI patients, and the lower panel shows the subgroups of anterior MI patients. All Δ are calculated after logarithmic transformations. Correlation coefficients are reported as Pearson linear correlations. (PDF 806 kb) [file 40168_2018_441_MOESM4_ESM.pdf]

**A**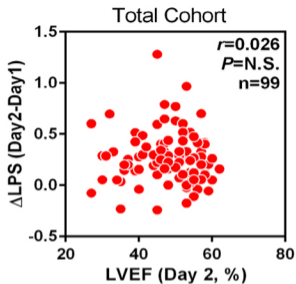**B**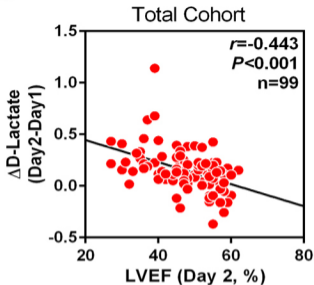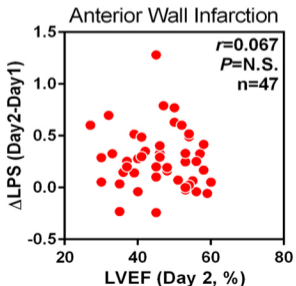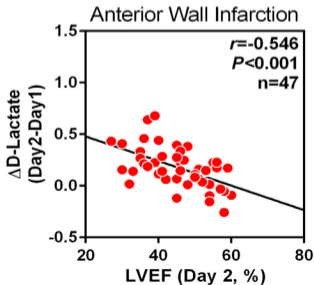

Supplement: Supplementary file 5 — Figure S3. The association of LPS, d-lactate with left ventricular ejection fraction (LVEF) after STEMI. (a) The relationship between Δ LPS and LVEF in all STEMI patients, and the subgroups of anterior MI patients, respectively. (b) Correlation analyses of Δ d-lactate and LVEF in total cohort and anterior MI subgroup. Correlation coefficients are reported as Pearson linear correlations. (PDF 387 kb) [file 40168_2018_441_MOESM5_ESM.pdf]

**a**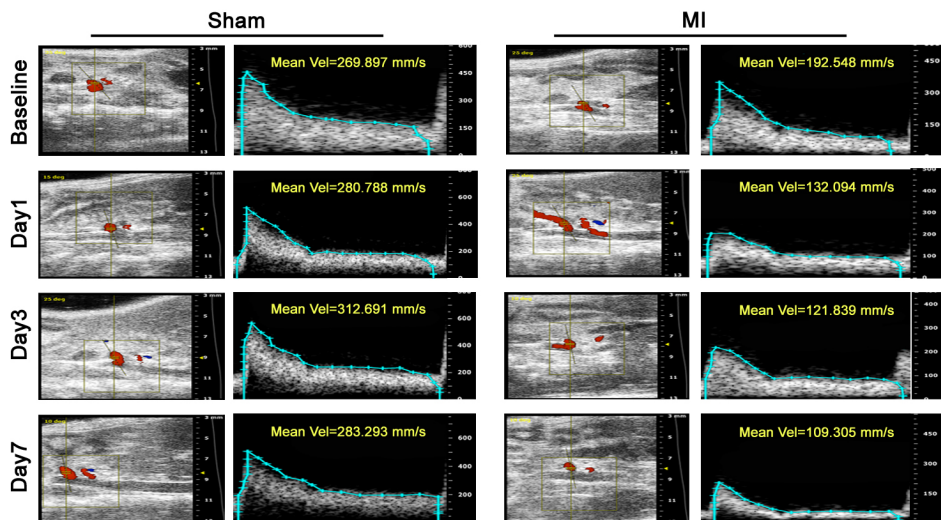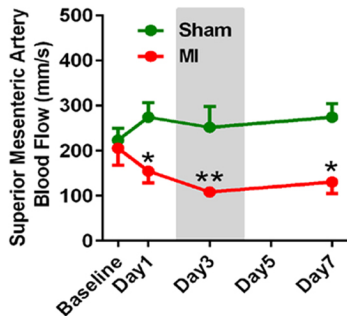**b**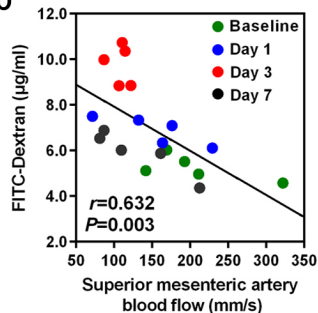**c**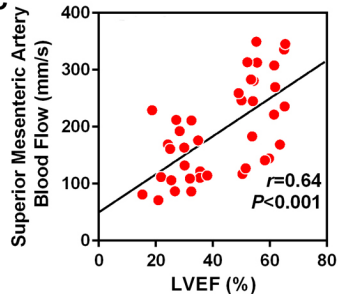

Supplement: Supplementary file 7 — Figure S5. The mesenteric artery blood flow in MI mice is associated with gut permeability and LVEF. (a) Echocardiographic measurements show significantly depressed blood flow of superior mesenteric artery post-infarction. n = 5 for each group. Data are presented as mean ± s.e.m. *P < 0.05, **P < 0.01 vs respective shams, t test. (b) The superior mesenteric artery blood flow is negatively correlated with gut permeability in the small intestine. (c) The association of LVEF with blood flow of superior mesenteric artery. (PDF 962 kb) [file 40168_2018_441_MOESM7_ESM.pdf]

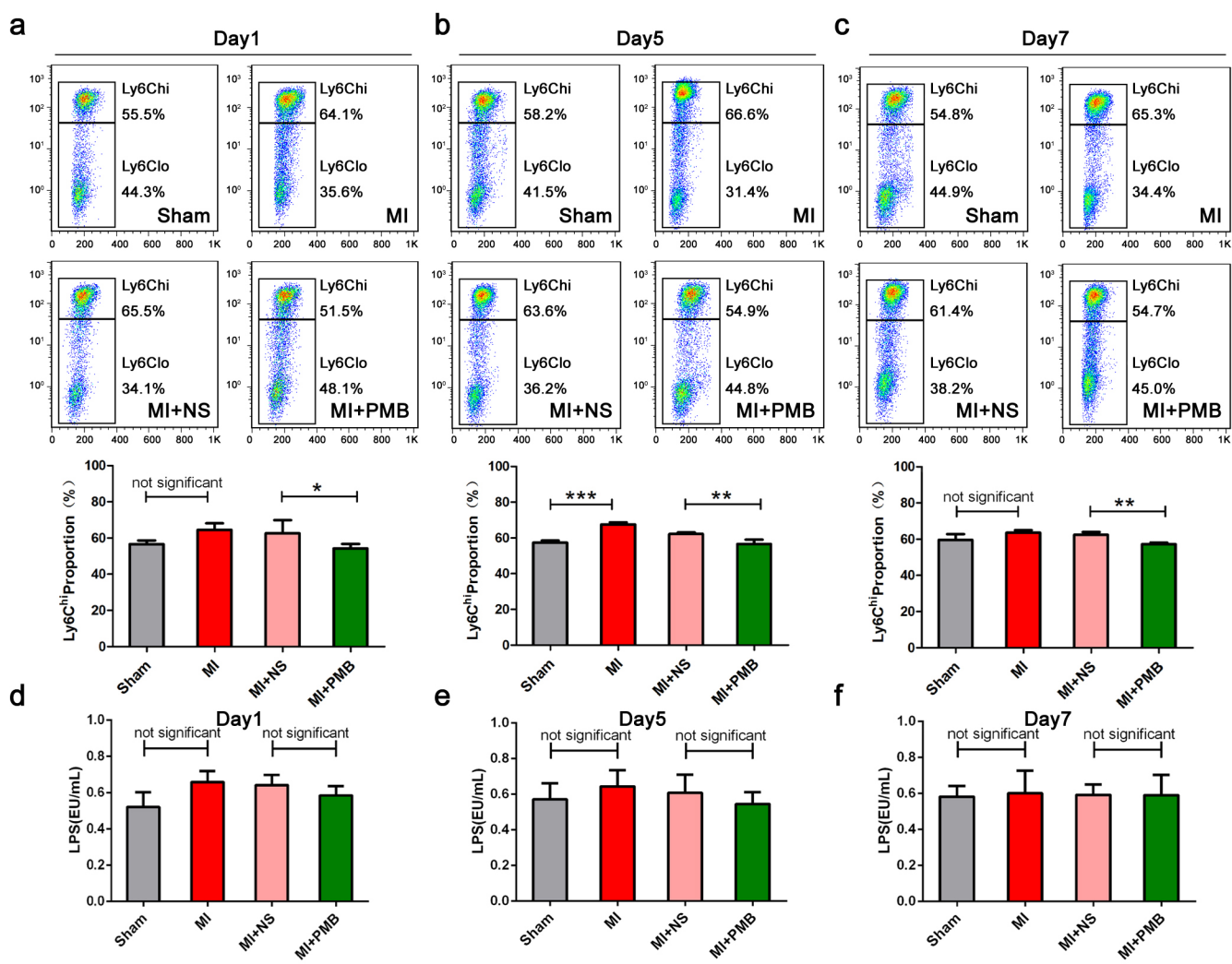

Supplement: Supplementary file 8 — Figure S6. The impact of PMB on LPS levels and Ly6Chi monocyte proportions. (a–c) PMB significantly represses the proportions of Ly6Chi monocyte at days 1, 5, and 7 post-infarction. n = 3–5 for each group. (d–f) There is no statistical significance of LPS levels by the treatment of PMB in mice of MI. n = 4–5 for each group. Data are presented as mean ± s.e.m. *P < 0.05, **P < 0.01, ***P < 0.001; one-way ANOVA followed by Tukey’s post hoc test. (PDF 1260 kb) [file 40168_2018_441_MOESM8_ESM.pdf]
