# Supplementary material for: Gut-dependent microbial translocation induces inflammation and cardiovascular events after ST-elevation myocardial infarction
Source: Microbiome. 2018 Apr 3;6:66. doi: 10.1186/s40168-018-0441-4 (PMC5883284; doi:10.1186/s40168-018-0441-4)
Supplement: Supplementary file 6 — Figure S4. The ROC curve of Δ LPS and Δ d-lactate for 3-year MACEs of STEMI patients. (PDF 686 kb) [file 40168_2018_441_MOESM6_ESM.pdf]

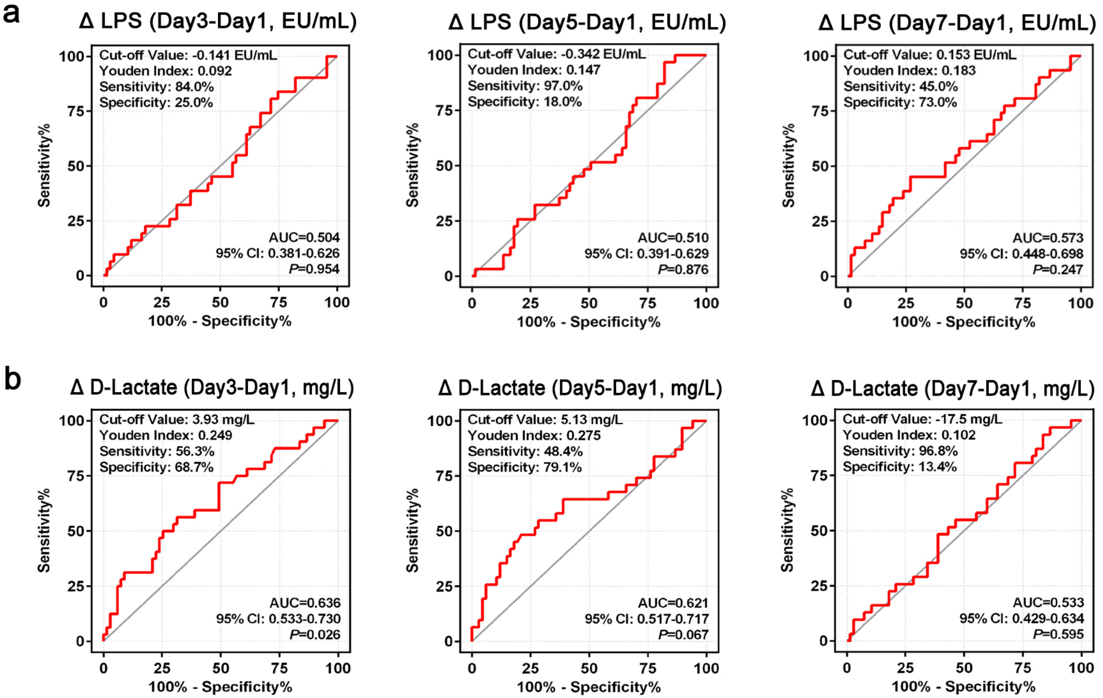
a
Δ LPS (Day3-Day1, EU/mL)
Cut-off Value: -0.141 EU/mL
Youden Index: 0.092
Sensitivity: 84.0%
Specificity: 25.0%
AUC=0.504
95% CI: 0.381-0.626
P=0.954
Δ LPS (Day5-Day1, EU/mL)
Cut-off Value: -0.342 EU/mL
Youden Index: 0.147
Sensitivity: 97.0%
Specificity: 18.0%
AUC=0.510
95% CI: 0.391-0.629
P=0.876
Δ LPS (Day7-Day1, EU/mL)
Cut-off Value: 0.153 EU/mL
Youden Index: 0.183
Sensitivity: 45.0%
Specificity: 73.0%
AUC=0.573
95% CI: 0.448-0.698
P=0.247
b
Δ D-Lactate (Day3-Day1, mg/L)
Cut-off Value: 3.93 mg/L
Youden Index: 0.249
Sensitivity: 56.3%
Specificity: 68.7%
AUC=0.636
95% CI: 0.533-0.730
P=0.026
Δ D-Lactate (Day5-Day1, mg/L)
Cut-off Value: 5.13 mg/L
Youden Index: 0.275
Sensitivity: 48.4%
Specificity: 79.1%
AUC=0.621
95% CI: 0.517-0.717
P=0.067
Δ D-Lactate (Day7-Day1, mg/L)
Cut-off Value: -17.5 mg/L
Youden Index: 0.102
Sensitivity: 96.8%
Specificity: 13.4%
AUC=0.533
95% CI: 0.429-0.634
P=0.595
Sensitivity%
100% - Specificity%
0
25
50
75
100
