# Supplementary material for: Gut-dependent microbial translocation induces inflammation and cardiovascular events after ST-elevation myocardial infarction
Source: Microbiome. 2018 Apr 3;6:66. doi: 10.1186/s40168-018-0441-4 (PMC5883284; doi:10.1186/s40168-018-0441-4)
Supplement: Supplementary file 9 — Supplementary methods. The script and code used for microbiota analysis. (PDF 41 kb) [file 40168_2018_441_MOESM9_ESM.pdf]

Script for microbiota analysis

Using QIIME to analyze 16S rRNA gene sequences

```
## step1: quality filtering(Shell) ###
flash sampleA.raw_data _1.fq.gz sampleA.raw_data _2.fq.gz -o raw_data/A/
python split_libraries_fastq.py -i ./raw_data/A/A.extendedFrags.fastq -o ./raw_data/A -m ./raw_data/A/A.mf -q 19 --barcode_type not-barcoded --sample_id A
# for other samples, the script is same with sampleA
cat raw_data/*/*.fna >raw_data/seq.fna
## step2: OTUs picking and annotation(Shell) ##
python pick_otus.py -i raw_data/seq.fna -o otus/uclust_picked_otus
python pick_rep_set.py -i otus/uclust_picked_otus/seq_otus.txt -f raw_data/seq.fna -l otus/rep_set/seq_rep_set.log -o otus/rep_set/seq_rep_set.fasta
python assign_taxonomy.py -i otus/rep_set/seq_rep_set.fasta -o otus/rdp_assigned_taxonomy --reference_seqs_fp GreenGene.97_otus.fasta --id_to_taxonomy_fp GreenGene.97_otu_taxonomy.txt
python make_otu_table.py -i otus/uclust_picked_otus/seq_otus.txt -t otus/rdp_assigned_taxonomy/seq_rep_set_tax_assignments.txt -o otus/otu_table.biom
python   single_rarefaction.py -i otus/otu_table.biom -o otus/otu_table_even.biom -d seq.num
python   summarize_taxa.py -i otus/otu_table_even.biom -o taxa --suppress_biom_table_output
python   align_seqs.py -i otus/rep_set/seq_rep_set.fasta -o otus/pynast_aligned_seqs
```

```
python    filter_alignment.py -i
otus/pynast_aligned_seqs/seq_rep_set_aligned.fasta -o
otus/pynast_aligned_seqs/
python    make_phylogeny.py -i
otus/pynast_aligned_seqs/seq_rep_set_aligned_pfiltered.fasta -o
otus/rep_set.tre
## step3: alpha diversity(Shell) ##
python alpha_diversity.py -i otus/otu_table_even.biom -m
observed_species,shannon,chao1 -o alpha_diversity/alpha_div.txt
## step4: beta diversity(Shell) ##
python beta_diversity.py -i otus/otu_table_even.biom -o beta_div -t
otus/rep_set.tre
### end ###
```

###### for figure 1-a ######

```
### begin ###
## step1: drawing a picture(R) ##
data <- read.table("OTU.Stat.For.Samples.txt",header=F,sep="\t")
# OTU.Stat.For.Samples.txt: SamplesID, OTU, Group
data <- data.frame(data)
p <- ggplot(data,aes(x=Group,y=OTU,fill=Group))
p + scale_fill_manual(values=c("#00c000","#0000ff","#ff0000")) +
geom_violin() +xlim( "Control", "CHD",
"STEMI")+labs(x='',y='Number of
OTUs',main="")+geom_boxplot(aes(x=Group,y=OTU),width=0.08,fi
ll="white",size=1)+stat_summary(fun.y="median",
geom="point",color="black",size=4)
## step2: Wilcoxon rank sum test(R) ##
Control <- data[data$Group == "Control",]
CHD <- data[data$Group == "CHD",]
STEMI <- data[data$Group == "STEMI",]
```

```
wilcox.test(CHD$OTU,STEMI$OTU,alternative="greater")
# p-value = 2.572e-06
wilcox.test(Control$OTU,STEMI$OTU,alternative="greater")
# p-value = 0.0907
### end ###
```

###### for figure 1-b, the script is the same with figure 1-a ######

###### for figure 1-c ######

```
### begin ###
## step1: PCoA analysis(R) ##
data <- read.table("unweighted.unifrac.distance.matrix")
d <-   as.dist(data)
pco <- dudi.pco(d scannf=F, nf=2)
pc1 <- round((pco$eig/sum(pco$eig))*100,2)[1]
pc2 <- round((pco$eig/sum(pco$eig))*100,2)[2]
write.csv(pco$li,file="PCoA.csv")
## step2: get PCoA.plot.txt(Shell) ##
perl -ne
'BEGIN{open(OR,"group.txt");while(<OR>){chomp;my@or=split/\s
+/;$hash{$or[0]}=$or[1];}close
OR;%col=("STEMI","STEMI","Control","Control","CHD","CHD");}ch
omp;my@or=split/,/;$or[0]=~s/"//g;print
"$hash{$or[0]}\t$or[1]\t$or[2]\t\"$col{$hash{$or[0]}}\"\n" if
$col{$hash{$or[0]}};'   PCoA.csv > PCoA.plot.txt
## step3: drawing a picture(R) ##
data <- read.table("PCoA.plot.txt",header=F)
colnames(data) <- c("SampleID","PC1","PC2","Group")
data <- data.frame(data)
p <-
```

```
ggplot(data,aes(x=PC1,y=PC2,col=Group,shape=factor(data$Grou
p)))
p  +scale_colour_manual(values=c("#ff0000","#00c000","#0000ff"))
+scale_shape_manual(values=c(19,17,15))  +  geom_point(size=2)
+labs(x='PC1(8.64%)',y='PC2(6.62%)')
## step4: Anosim & MRPP(R) ##
library(vegan)
otu <-   read.table("otu_table.txt",header=T)
otu <-   t(otu)
# Anosim & MRPP between STEMI and CHD
compareOTU <-   otu[c(1:99,100:149),]
group <-   c(rep("STEMI",99),rep("CHD",50))
otu.dist <-   vegdist(compareOTU)
otu.anosim <-   anosim(otu.dist,group)
otu.mrpp <-   mrpp(compareOTU,group,distance = "bray")
# Anosim & MRPP of Control-STEMI and Control-CHD are the same
with STEMI-CHD
### end ###
```

###### for figure 1-d ######

```
### begin ###
## step1: Wilcoxon rank sum test(R) ##
bacteriumInGroupA <- c(abundanceInGroupA)
bacteriumInGroupB <- c(abundanceInGroupB)
p <- wilcox.test(bacteriumInGroupA, bacteriumInGroupB)
p$p.value
## step2: Correcting Pvalues for multiple testing with Benjamin &
Hochberg(R) ##
p <- c(Pvalues) # p values that < 0.05
p.adjust(p, method = "fdr", n = length(p))
# different genera: adjusted p value < 0.05
```

There were 210 genera that displayed significant abundance differences in STEMIs as compared to controls and CHDs
end

###### for figure 1-e ######

begin
step1: drawing a picture of the top 40 most different genera across groups

```
library(pheatmap)
data <- read.table("diff.genus.top40.txt",sep="\t",header=T,row.names=1)
data <- data.frame(data)
pheatmap(data,cluster_cols=F,cluster_rows=T,fontsize=5,scale="row")
```

end

###### for figure 1-f ######

begin
step1: From HMP database, using the reference genomes isolated from human gut and oral to tracking the source of the STEMI-enriched bacteria

URL: http://www.hmpdacc.org/catalog/grid.php?dataset=genomic

step2: Based on NCBI taxonomy database, we can get the reference genomes' taxonomic and phylogenic information

URL: http://www.ncbi.nlm.nih.gov/Taxonomy/Browser/wwwtax.cgi

step3: based on the phylogenic information and the genera profile, the source of the genera was defined and the proportion of different groups was calculated(Perl)

```
use strict;
use warnings;
```

```
die "usage:perl $0 <input1:genera profile> <input2:source info>
<output>\n" unless @ARGV == 3;
my %tax2g;
open(OR,$ARGV[1]);
while(<OR>){
	next if /^#/;chomp; my@or=split/\t/;$tax2g{$or[0]}=$or[1];
}
close OR;
open(OR,"$ARGV[0]");
my %sample2g2abun;
my $head=<OR>;
chomp$head;
my@head=split/\t/,$head;
while (<OR>) {
    chomp;
    my @or=split/\t/;
    for(my $i = 1; $i <= $#head;$i++){
        next if ! $tax2g{$or[0]};
        $sample2g2abun{$head[$i]}{$tax2g{$or[0]}} += $or[$i];
    }
}
close OR;
open(OUT,">$ARGV[2]");
my $i ;
foreach my $g (sort keys %sample2g2abun){
	my @groups=sort keys %{$sample2g2abun{$g}};
	my @head=@groups;
	map{$_=~s/;/-/g;} @head;
	print OUT "\t".join("\t",@head)."\n" if ! $i ;
	print OUT "$g";
	foreach my $h (@groups){
```

```
            print OUT "\t$sample2g2abun{$g}{$h}";
        }
        print OUT "\n";
        $i=1;
}
close OUT;
### end ###
```

###### for figure-S1 ######

```
### begin ###
## step1: for Proteobacteria(R) ##
Abundance <- AbundanceInGroups
table <- data.frame(Abundance,Group)
table$Group <- factor(table$Groups, levels = c("Control","CHD","STEMI"))
ggplot(table,aes(y=Abundance,fill=Group,x=Group)) + scale_fill_manual(values=c("#00c000","#0000ff","#ff0000"))+geom_boxplot(notchwidth=1,outlier.size=0.5,colour="#525252")+labs(x=' ',y="Proteobacteria")
## for other phylum, the script is the same with Proteobacteria ##
### end ###
```
